# Don’t worry, it won’t be fine. Contributions of worry and anxious arousal to startle responses and event-related potentials in threat anticipation

**DOI:** 10.3758/s13415-023-01094-4

**Published:** 2023-04-27

**Authors:** Hannes Per Carsten, Kai Härpfer, Brady D. Nelson, Norbert Kathmann, Anja Riesel

**Affiliations:** 1 Department of Psychology, University of Hamburg, Von-Melle-Park 11, 20146 Hamburg, Germany; 2 Department of Psychology, Stony Brook University, Stony Brook, NY USA; 3 Department of Psychology, Humboldt University of Berlin, Berlin, Germany

**Keywords:** Anxiety dimensions, Startle reflex, Event-related potentials, NPU-threat test, Anxious apprehension, Threat anticipation, Uncertainty, Unpredictability

## Abstract

A widely shared framework suggests that anxiety maps onto two dimensions: anxious apprehension and anxious arousal. Previous research linked individual differences in these dimensions to differential neural response patterns in neuropsychological, imaging, and physiological studies. Differential effects of the anxiety dimensions might contribute to inconsistencies in prior studies that examined neural processes underlying anxiety, such as hypersensitivity to unpredictable threat. We investigated the association between trait worry (as a key component of anxious apprehension), anxious arousal, and the neural processing of anticipated threat. From a large online community sample (*N* = 1,603), we invited 136 participants with converging and diverging worry and anxious arousal profiles into the laboratory. Participants underwent the NPU-threat test with alternating phases of unpredictable threat, predictable threat, and safety, while physiological responses (startle reflex and startle probe locked event-related potential components N1 and P3) were recorded. Worry was associated with increased startle responses to unpredictable threat and increased attentional allocation (P3) to startle probes in predictable threat anticipation. Anxious arousal was associated with increased startle and N1 in unpredictable threat anticipation. These results suggest that trait variations in the anxiety dimensions shape the dynamics of neural processing of threat. Specifically, trait worry seems to simultaneously increase automatic defensive preparation during unpredictable threat and increase attentional responding to threat-irrelevant stimuli during predictable threat anticipation. The current study highlights the utility of anxiety dimensions to understand how physiological responses during threat anticipation are altered in anxiety and supports that worry is associated with hypersensitivity to unpredictable, aversive contexts.

**Supplementary Information:**

The online version contains supplementary material available at 10.3758/s13415-023-01094-4.

## Introduction

Anxiety disorders are prevalent (Baxter et al., 2013), highly comorbid (Kessler et al., 2005; Stein et al., 2017), and impose an immense burden of disease on the affected individuals (Baxter et al., 2014; Wittchen et al., 2011). This underscores the importance of studying the underlying mechanisms that contribute to the development and maintenance of anxiety disorders. Yet, shortcomings emerge from studies trying to identify these underlying mechanisms of anxiety by using categorical approaches (Lang et al., 2016; Marin et al., 2020). Comorbidity is often unattended to and findings are rarely disorder-specific (Lang et al., 2016). This calls for approaches that examine transdiagnostic symptom dimensions to study anxiety symptomatology, rather than categorical and disorder-specific analyses. Recent transdiagnostic research endeavors guided by the principles of the Research Domain Criteria (RDoC; Insel et al., 2010) have begun to illuminate the underlying mechanisms of more specific transdiagnostic phenotypic expressions of anxiety (Duits et al., 2015; Janiri et al., 2020; Lang et al., 2016; Sharp et al., 2015), such as hypersensitivity to unpredictable threat (Gorka et al., 2017). However, how the underlying mechanisms of anxiety disorders manifest themselves across units of analysis (e.g., self-report and psychophysiology; Cuthbert, 2014) remains vague. Unraveling the interplay of the underlying mechanisms of anxiety across units of analysis could be used to tailor interventions to individual anxiety symptom profiles (Sharp et al., 2015). Ultimately, this could help to alleviate the burden of disease generated by anxiety disorders.

Among various frameworks modelling anxiety symptomatology, one largely shared decomposition of anxiety symptomatology proposes two psychologically and neurally distinct dimensions (Cox et al., 2010; Heller et al., 1997; Lang et al., 2016; Sharp et al., 2015). First, *anxious apprehension*—a trait that can condense into an emotional state of anxiety and symptoms of worry—describes the tendency for verbal, future-oriented rumination characterized by a perceived inability to predict, control, or cope with the anticipated events (Barlow, 1991; Heller et al., 1997). Second, the trait *anxious arousal*—corresponding to emotional states of panic and fear—captures the propensity to experience symptoms of physiological hyperarousal and somatic tension when faced with even mildly threatening stimuli (Clark and Watson, 1991; Sharp et al., 2015; Watson et al., 1995). Anxious apprehension and anxious arousal are distinguishable but not distinct dimensions (Engels et al., 2007). They co-occur (Sharp et al., 2015) but differ in their clinical manifestations. Anxious apprehension is phenotypically predominant in patients with obsessive-compulsive disorder and patients with generalized anxiety disorder (Cox et al., 2010; Krueger, 1999). Anxious arousal is more pronounced in focal fears (e.g., specific phobia; Lang et al., 2016). The anxiety dimensions of anxious apprehension and anxious arousal could provide a theoretical framework that maps different phenotypic expressions of anxiety disorders. Considering the anxiety dimensions as a theoretical framework in studies could aid with integrating heterogeneous findings across anxiety-disorder categories and facilitate to reveal the underlying mechanisms. Anxious apprehension and anxious arousal are primarily measured via self-report (Sharp et al., 2015). In the current study, we operationalized anxious apprehension via the central symptom component worry as a trait-variable (Glöckner-Rist and Rist, 2014; Meyer et al., 1990; Sharp et al., 2015). We aimed at testing the correlates of self-reported individual differences in worry and anxious arousal in peripheral physiology and neural processing (Sharp et al., 2015).

Therefore, we examined associations between worry, anxious arousal, and responses to the anticipation of predictable and unpredictable threat relative to safety by using the NPU-threat test (Grillon et al., 2004; Nelson and Shankman, 2011; Schmitz and Grillon, 2012). The NPU-threat test enables differentiating responses to temporally predictable threat from responses to temporally unpredictable threat. In the neutral condition, participants never receive an aversive stimulus. In contrast, in the predictable condition, the participants receive a temporally predictable aversive stimulus signaled by a cue. Responses in the predictable condition are closely linked to the emotional states of fear (i.e., phasic response to predictable imminent threat; Schmitz and Grillon, 2012). In the unpredictable condition, participants may receive an unsignaled, aversive stimulus at any time. Responses in the unpredictable condition are thought to reflect emotional states of anxiety and defensive preparedness (i.e., sustained tension due to the unpredictability of threat; Schmitz and Grillon, 2012). Thus, the NPU-threat test enables investigating possible links between worry and anxious arousal and the corresponding states of anxiety (unpredictable threat condition) and fear (predictable threat condition).

Previous studies using the NPU-threat test mostly measured the eyeblink component of the startle reflex (Lieberman, Stevens, et al., 2017b; Nelson and Shankman, 2011; Schmitz and Grillon, 2012). The startle reflex captures defensive responding (Blumenthal et al., 2005; Hamm et al., 1993), is initiated in the nucleus reticularis pontis caudalis, and is modulated by affective information from the centromedial amygdala (Kuhn et al., 2020). Compared with the neutral condition, startle responses in the unpredictable condition are usually increased (anxiety-potentiated). To a lesser extent, startle responses in the predictable condition are usually increased compared with the neutral condition (fear-potentiated; Bradford, Kaye, and Curtin, 2014; MacNamara and Barley, 2018; Schmitz and Grillon, 2012). Fear-potentiated startle might be sensitive to the modality of the aversive stimulus, as Ferry and Nelson (2020) found decreased startle in anticipation of predictable screams compared with shocks. To capture the temporal dynamics of the neural processing of threat stimuli, previous NPU-studies used the event-related potential (ERP) components N1 and P3 (Carsten et al., 2022; Ferry and Nelson, 2020; MacNamara and Barley, 2018; Nelson, Hajcak, et al., 2015a; Nelson, Hodges, et al., 2015b; Stevens et al., 2018). The N1 is observable as a frontocentral negative deflection approximately 100 ms after stimulus onset. Locked to acoustic startle probes (i.e., probe locked), the N1 likely reflects early sensory processing (Cuthbert et al., 1998) in auditory cortical areas (Ford et al., 2016; Verkindt et al., 1995). The P3 is observable as a positive centroparietal deflection approximately 300 ms after stimulus onset and likely reflects attentional allocation at a later stage of the information-processing cascade (Polich, 2012). Subcomponents of the P3 are theorized to originate from frontal lobe activation (P3a) and tempoparietal activation, respectively (P3b; Polich, 2007, 2012). Previous studies found elevated probe locked N1 specific to the unpredictable condition of the NPU-threat test (Ferry and Nelson, 2020; Nelson, Hajcak, et al., 2015a; Nelson and Hajcak, 2017; but not in MacNamara and Barley, 2018). These studies also found evidence for a suppressed probe locked P3 in the unpredictable condition and predictable condition relative to the neutral condition, which presumably reflects attentional allocation to the task rather than to the startle probe (Ferry and Nelson, 2020; MacNamara and Barley, 2018; Nelson, Hajcak, et al., 2015a; Nelson and Hajcak, 2017; but see Stevens et al., 2018). Taken together, the probe locked N1 seems to be more closely linked to unpredictable threat anticipation, whereas the probe locked P3 might be insensitive to threat predictability (Nelson, Hajcak, et al., 2015a). In the present study, we included startle and probe locked N1 and P3 to elucidate different aspects and the temporal dynamics of threat processing and to examine their association to individual differences in trait worry and anxious arousal.

Previous research on possible connections between anxiety dimensions and the neural basis of threat processing is scarce. We derived indications from theoretical reasoning as well as from clinical and subclinical studies focusing on the anxiety dimensions. From a theoretical perspective, anxious apprehension is linked to states of worry (Sharp et al., 2015), which in turn is considered a coping strategy for unpredictability and uncertainty (Einstein, 2014; Sibrava and Borkovec, 2006). Thus, unpredictable situations might be more aversive for individuals with increased trait worry (Carleton, 2016b; Einstein, 2014). This increased aversion, in turn, could manifest itself in increased responses to unpredictable threat. In contrast, anxious arousal corresponds to physiological hyperreactivity (Sharp et al., 2015), which is associated with more focal fears (specific phobia; Lang et al., 2016). Based on this, increased physiological responses to predictable threat could be expected as a function of anxious arousal. A clinical study of anxiety disorders characterized by worry (generalized anxiety disorder; Cox et al., 2010; Goodwin et al., 2017; Krueger, 1999) found on a trend level elevated startle responses in the unpredictable condition (Grillon et al., 2017). In contrast, other studies did not find an effect of generalized anxiety disorder on startle responses (Gorka et al., 2017; Grillon et al., 2009). Regarding disorders predominantly characterized by anxious arousal, elevated startle responses to unpredictable but not predictable threat were found in patients with specific phobia (Gorka et al., 2017). This contradicts the above described theoretically derived assumption that anxious arousal might be insensitive to threat predictability. Dimensional studies following the RDoC principles (Insel et al., 2010) focus on individual differences in anxiety-related traits. Whereas Nelson and Shankman (2011) found no significant associations of worry and startle responses in the unpredictable condition in a nonclinical sample, Rutherford et al. (2020) found this association specifically in individuals with a history of anxiety disorders. Previous studies associated cognitive concerns—a trait that is linked to worry (Wheaton et al., 2012, but cf. Olthuis et al., 2014)—with attenuated startle responses in the unpredictable condition (Nelson, Hodges, et al., 2015b), but no evidence emerged for probe locked N1 or P3 (Stevens et al., 2018). To the best of our knowledge, individual differences in self-reported anxious arousal have not yet been tested in the context of the NPU-threat test. In summary, the delineated findings from clinical and subclinical studies are contradictory, thereby impede precise predictions, and thus highlight the need for further research. Furthermore, prior studies have not directly examined worry and anxious arousal simultaneously. Thus, the heterogeneity of findings in clinical studies might be attributable to individual differences in these transdiagnostic anxiety dimensions. Yet, we lack knowledge on the isolated effects and possible interactions of these dimensions. Because of the heterogeneity of theoretical predictions and mixed empirical findings, we did not formulate directional hypotheses regarding links of worry and anxious arousal to physiological responses in the NPU-threat test.

The present study used self-report measures of worry and anxious arousal, the NPU-threat test, and physiological outcome measures. This aims to elucidate whether worry and anxious arousal influence physiological responses (startle and ERP components N1 and P3) in the anticipation of threat. Examining both anxiety dimensions simultaneously allows us to test possible interaction effects of the anxiety dimensions on threat anticipation. This might advance our understanding of how anxious apprehension and anxious arousal, as central dimensions of anxiety, influence the temporal dynamics of the neural processing of aversive stimuli in the context of predictable and unpredictable threat.

## Methods

### Participants

**Fig. 1:**
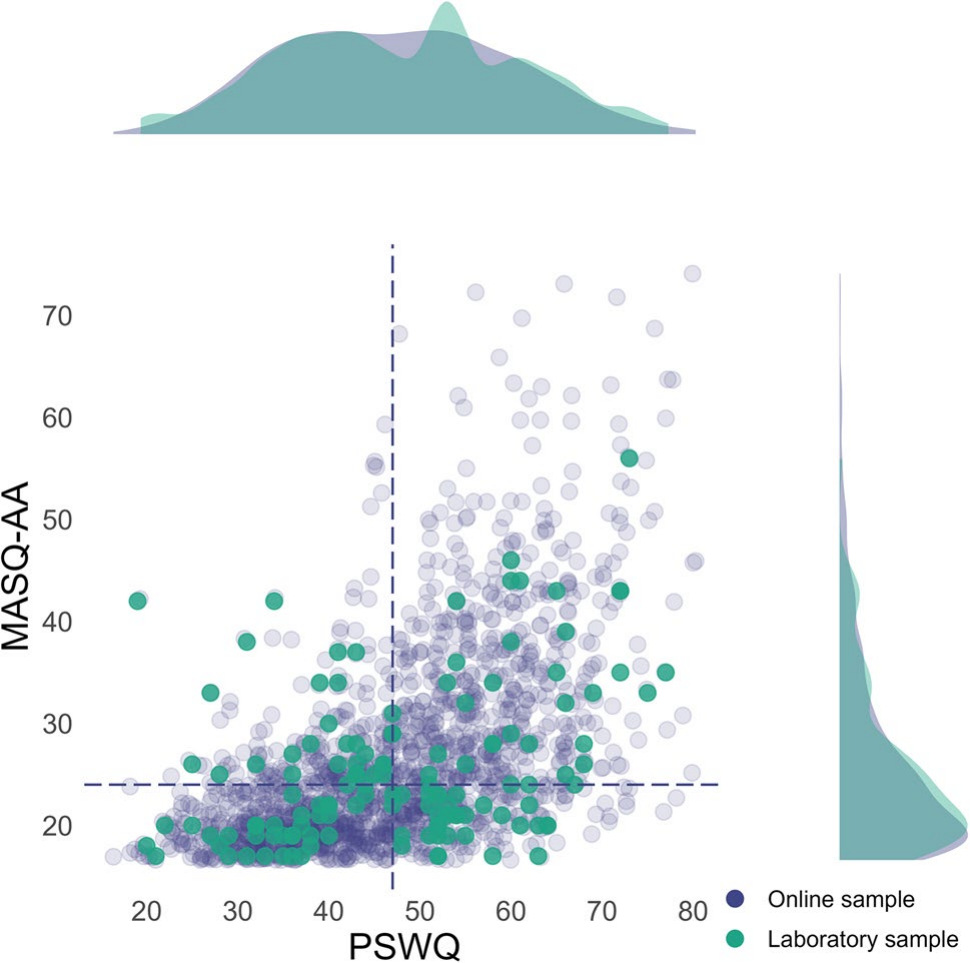
Scatter plots and density distribution of PSWQ and MASQ-AA scores. *Note*. From an online screening sample (*N* = 1,603), we invited *N* = 136 participants to the laboratory to perform the NPU-threat test while we collected psychophysiological data. The recruitment was based on the median of the questionnaires operationalizing the anxiety dimensions, i.e., Penn State Worry Questionnaire (PSWQ, *Mdn* = 47, dashed vertical line) and the anxious arousal subscale of the Mood and Anxiety Symptom Questionnaire (MASQ-AA, *Mdn* = 24, dashed horizontal line). To obtain equal sample sizes in each group of the median based 2 (PSWQ: high vs. low) × 2 (MASQ-AA: high vs. low) design, we oversampled participants in whom one dimension was more pronounced than the other

An online screening sample (*N* = 1,603, 72.5% female, 27.5% male, aged 18-65, *M =* 29.06, *SD =* 9.29) was recruited via university mailing lists and social networks. The online screening sample only completed questionnaires capturing individual differences in the anxiety dimensions, i.e., the Penn State Worry Questionnaire (PSWQ; 16 items, 5-point Likert scale 1-5, “not at all typical of me” to “very typical of me”; *N* = 1,603; Glöckner-Rist and Rist, 2014; Meyer et al., 1990) to measure trait worry and the anxious arousal subscale of the Mood and Anxiety Symptom Questionnaire (MASQ-AA; 17 items, 5-point Likert scale 1-5, *N* = 1,603; Watson and Clark, 1991). To perform the NPU-threat test, we invited 136 participants from the online screening sample into the laboratory (Fig. 1). These participants were selected from the online screening sample based on their PSWQ and MASQ-AA values. Specifically, we formed a 2 (PSWQ: high vs. low) × 2 (MASQ-AA: high vs. low) design based on median splits of the online screening sample (PSWQ *Mdn* = 47, MASQ-AA *Mdn* = 24; Härpfer et al., 2020; Kausche et al., 2022). During recruiting—to obtain equal sample sizes in each group of the median based 2×2 design—we oversampled participants from the online sample in whom one dimension was more pronounced than the other. This optimizes the variance distribution and facilitates disentangling possible interaction effects (Smith et al., 2016; Zambrano-Vazquez and Allen, 2014) but does not result in distinct groups (Fig. 1); therefore, we analyzed the data dimensionally. The sampling strategy lowered the correlation between PSWQ and MASQ-AA from *r* = 0.56 (online screening sample, *p* < .001) to *r* = 0.36 (laboratory sample, *p* < .001). The age of the 136 participants ranged from 18–62 years (*M* = 28.78, *SD* = 9.50), 63% of the participants identified as female and 37% identified as male.

Exclusion criteria encompassed lifetime neurological diseases, bipolar disorder, schizophrenia spectrum disorder, or substance-related disorder assessed via clinical screening (Wittchen et al., 1997). Participants were further excluded for the intake of benzodiazepines 1 week before testing or the intake of neuroleptics 3 months before testing, assessed via self-report. Participants gave written, informed consent before participation. The ethics committee of the Humboldt-Universität zu Berlin approved the study as being in accordance with the Declaration of Helsinki (World Medical Association, 2013).

### Questionnaires

In the online sample, the PSWQ (*M* = 47.10, *SD* = 12.46, *N* = 1,603) showed good internal consistency (α = 0.93), akin to the MASQ-AA (*M* = 26.57, *SD* = 9.59, α = 0.90). In the laboratory sample, the PSWQ scores ranged from 19-77 (*M* = 47.62, *SD* = 13.28), the MASQ-AA scores ranged from 17–56 (*M* = 25.79, *SD* = 8.12; Fig. 1). In addition to the PSWQ and MASQ-AA, the laboratory sample completed the State-Trait-Anxiety Inventory measuring trait anxiety (STAI-T; *M* = 38.70, *SD* = 9.25, 20 items, 4-point Likert scale, 1-4, α = 0.92, *N* = 136; Spielberger et al., 1983; Laux, 1981) and the Obsessive-Compulsive Inventory Revised measuring obsessive-compulsive symptoms (OCI-R; *M* = 12.50, *SD* = 9.75, 20 items, 5-point Likert scale 0-4, α = 0.88, *N* = 136; Foa et al., 2002; Gönner et al., 2007). Furthermore, depressive symptoms were measured using the Beck Depression Inventory (BDI-II; *M* = 7.71, *SD* = 7.15, 21 items, 4-point Likert scale 0-3, α = 0.89, *N* = 136; Beck et al., 1996; Hautzinger et al., 2006).

### Task and stimuli

**Fig. 2:**
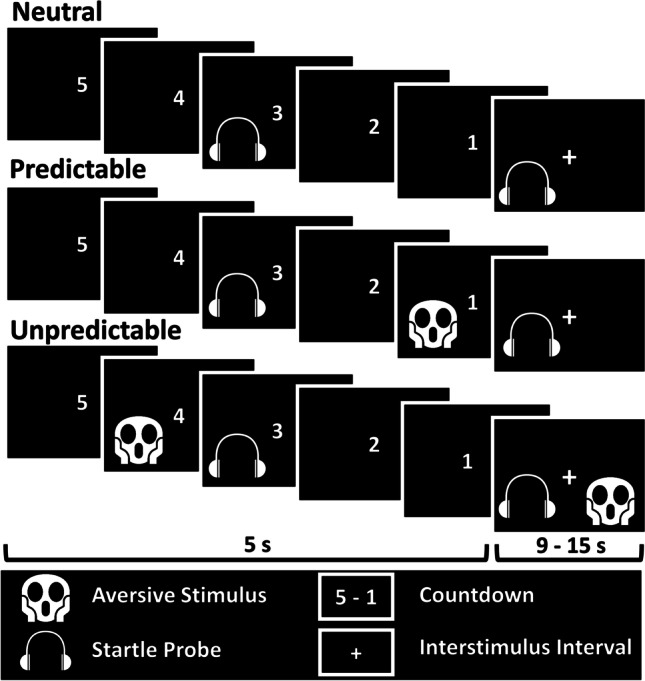
NPU-threat test. *Note.* In the neutral condition, the countdown was never followed by the aversive stimulus (100 dB, auditory scream paired with the picture of a fearful face, image 01F from NimStim Tottenham et al. (2009)). In the predictable condition, the aversive stimulus occurred every time the countdown reached “1”. In the unpredictable condition, the aversive stimulus could occur at any time during the countdown or the interstimulus interval

The participants completed the task in a dimly lit cabin, shielded from auditory and electromagnetic noise. Visual stimuli were presented on a 19-inch LCD monitor (Dell, 1907 FPV, 1,280 x 1,024 pixels, 60-Hz refresh rate) in a viewing distance of approximately 65 cm using Presentation Software version 20.2 (Neurobehavioral Systems, Inc., Albany, California). We used the countdown version of the NPU-threat test (Nelson and Shankman, 2011; Schmitz and Grillon, 2012; Fig. 2). Each condition (i.e., neutral, predictable, unpredictable) was presented twice, resulting in two orders (PNUPNU or UNPUNP) which were randomized across participants. A numerical countdown counting from five to one served as the threat cue (1 s per digit), and a black screen stating the current condition served as the interstimulus interval (i.e., cue offset to the onset of the subsequent cue, 12 ± 3 s). Each condition included 9 cues (i.e., 5 interstimulus intervals and 4 countdowns) and a black screen (10 s) separated the conditions. A female scream delivered through speakers in front of the participant (100 dB, 1 s) paired with the picture of the face of a fearful woman served as the aversive stimulus (NimStim image 01F; Tottenham et al., 2009). Four aversive stimuli occurred per condition (i.e., predictable and unpredictable). In the predictable condition, the aversive stimulus occurred when the countdown reached “1” (in 100% of the presentations), but not during the presentation of the interstimulus interval. In the unpredictable condition, the aversive stimulus could occur at any time during the interstimulus interval (5.5 ± 2.5 s, in 40% of the presentations) and countdown (4.2 ± 0.5 s, in 50% of the presentations). Thus, the temporal predictability of the aversive stimulus was manipulated. Headphones delivered startle probes to elicit the startle reflex in 75% of the interstimulus intervals and countdowns. The startle probes consisted of 93 dB bursts of white noise with a duration of 50 ms (rise/fall time < 1 ms). Before the experiment, the participants received 6 acoustic startle probes to allow an initial startle habituation (Blumenthal et al., 2005).

### Startle recording and processing

The eyeblink component of the startle response was recorded as electromyographic (EMG) signal measuring the contraction of the left orbicularis oculi muscle. Therefore, we attached one Ag/AgCl electrode (sensor Ø = 4 mm) vertically in line with the pupil, as close to the palpebrae inferior as possible and placed another electrode 1.5 cm laterally at the same horizontal level.

EMG data were recorded using BrainVision Recorder 2.1 and a 16-channel BrainAmp amplifier (Brain Products GmbH, Gilching, Germany) with a sampling rate of 1,000 Hz and an online band-pass filter (0.01–1,000 Hz). EMG data were preprocessed and scored offline by using BrainVision Analyzer 2.2 (Brain Products GmbH, Gilching, Germany). A band-pass filter (28–500 Hz, 24 dB/octave roll off) was applied before rectification and baseline-correction (50 ms interval before startle probe onset). Subsequently, a moving average of 9 ms integrated the data. Amplitudes of the startle EMG signal were scored manually from onset to peak in a response latency window of 20 to 120 ms after startle probe onset. The response onset was defined as a visually distinguishable difference from the baseline (−50 to 20 ms relative to the startle probe). If a response onset was detected, the peak was defined as the highest first peak in a peak window 25 to 150 ms relative to the startle probe. If no reaction emerged in the response latency window, the segment was scored as zero and was included into the analyses (*M* = 33.64%, *SD* = 27.18%; Blumenthal et al., 2005). If a voluntary or spontaneous blink occurred in the interval −50 ms to 20 ms relative to the startle probe onset, the reaction was scored as missing and excluded from the analyses (*M* = 6.78%, *SD* = 5.69%; Blumenthal 2005). The single trial startle responses were entered into the analyses without aggregation (participant’s individual trials *M* = 33.66, *Mdn* = 34, *SD* = 2.00). Internal consistency of the startle responses was calculated by using Cronbach’s alpha based on single trials for each cue within each condition and ranged from α = 0.868 to α = 0.942. An independent rater blinded to the experimental conditions repeated the manual scoring. To determine interrater reliability, the data were aggregated per cue (countdown, interstimulus interval) in each experimental condition (neutral, predictable, unpredictable). Krippendorff’s α was calculated using the R package “krippendorffsalpha” (Hughes, 2021). Between the raters, the reliability was near perfect (α = 0.997, 95% confidence interval [CI] [0.996, 0.997]; Hughes, 2021).

### Electroencephalography recording and processing

Electroencephalographical (EEG) activity was measured with 60 concentric and equidistant Ag/AgCl scalp electrodes (Easycap, Herrsching, Germany) with additional external electrodes placed at the nasion, the neck, and the left infraorbital site. The ground electrode was positioned on the right cheek. Electrode Cz was the recording reference. All impedances were kept <5 kΩ. Continuous EEG signals were digitized at a sampling rate of 1,000 Hz with two 32-channel BrainAmp amplifiers (Brain Products GmbH, Gilching, Germany) and recorded with a band-pass filter of 0.1 to 250 Hz.

Raw data were preprocessed using the software Brain Vision Analyzer 2.2 (Brain Products GmbH, Gilching, Germany) and were digitally filtered with low and high cutoffs of 0.1 and 30 Hz, respectively, and a notch filter of 50 Hz. A semiautomatic, independent component analysis (ICA) was used to correct for vertical and horizontal eye movement artifacts. Before segmentation, electrodes were re-referenced to a common average reference. Subsequently, epochs of 1,000 ms starting at −200 ms relative to the startle probe onset were extracted. The epochs were baseline corrected (−200 ms to startle probe onset) after artifact rejection. Artifacts were defined as an absolute voltage range in a segment exceeding 300 μV, or a voltage step between consecutive data points exceeding 50 μV, or a voltage difference of less than 0.5 μV within a 100-ms interval (Nelson, Hajcak, et al., 2015; Nelson and Hajcak, 2017). Additionally, we visually inspected the data to detect and reject remaining artifacts. Artifact rejection caused an average data loss of 0.11% (*SD* = 0.38), i.e., on average, less than 1 of 36 trials per participant was contaminated with artifacts, and no participant was excluded from the EEG data analyses due excessive artifacts or equipment failure. The exclusion criterion was less than 50% usable trials in at least one experimental condition (i.e., neutral, predictable or unpredictable; Nelson, Hajcak, et al., 2015a; Nelson and Hajcak, 2017). Next, the epochs were averaged per cue (interstimulus interval, countdown) separately for each condition (neutral, predictable, unpredictable) within each participant. This aggregation left 6 data points per participant per ERP component (i.e., N1 and P3).

We scored the ERPs based on grand-averaged waveforms and topographical distribution (Supplement H: Figure S2): The probe N1 was maximal at electrode FCz between 120–170 ms and was quantified as ±25 ms area around the individual peak. The probe P3 was maximal at the electrode Pz and was scored as mean activity 300–370 ms after startle probe onset. To determine split half reliability, we calculated Spearman-Brown corrected correlations of odd and even trials for each condition (i.e., neutral, predictable, and unpredictable). For N1, these ranged from *r* = 0.88 to 0.90. For P3 these ranged from *r* = 0.61 (predictable condition) to *r* = 0.80 (neutral and unpredictable condition).

### Data analysis

We analyzed the data using R version 4.0.3 (R Core Team, 2020) and RStudio version 1.3.1093 (RStudio Team, 2020). A *p*-value of .05 formed the significance threshold. Multilevel regression models (MLM) were conducted for each outcome measure separately (startle, N1, P3) using the lme4 package (Bates et al., 2015). MLMs hold three advantages for the present data structure. First, the questionnaires (PSWQ and MASQ-AA) are analyzed continuously without forming artificial groups. Second, random effects can model individual differences in absolute amplitude. Raw EMG startle data require no standardization and variations in the within-person effects can be modelled using random effects (i.e., via random intercepts). Third, no data aggregation is necessary for the EMG data, which increases statistical power (Hox et al., 2017). Note, that data on ERPs were aggregated within each participant per cue per condition of the NPU-threat test because of low signal-to-noise ratio in single trial EEG data. The intraclass correlation (ICC) was estimated from the null models, which indicates the proportion of the total variance explained by the grouping structure in the population (Hox et al., 2017). The models were estimated by using maximum likelihood estimation and an unstructured covariance matrix. For the final models, the individual amplitudes of single trial startle and aggregated ERPs (level 1) to each cue and condition were nested within the respective participant (level 2). Fixed effects included the unweighted effect-coded level 1 (i.e., within participant) predictors condition (neutral, predictable, unpredictable) and cue (interstimulus interval, countdown). For condition, neutral was the reference category, for cue, interstimulus interval was the reference category. Individual PSWQ and MASQ-AA values were grand-mean centered and entered as level 2 (i.e., between participant) predictors. The predictors PSWQ and MASQ-AA were entered into the models together. Thus, any significant cross-level interaction involving PSWQ or MASQ-AA suggests specificity of that questionnaire relative to the other questionnaire. Semipartial *R*^*2*^ indicates the partial effect sizes for each model parameter (Edwards et al., 2008; Jaeger, 2017). A maximal random approach was used to model random effects (Barr et al., 2013). The random effects included random intercepts for each individual participant, as models with random slopes for condition failed to converge. Significant cross-level interactions, including PSWQ or MASQ-AA, were followed up by pairwise comparisons of the PSWQ or MASQ-AA slopes for each level of the interacting factorial predictor (Russell et al., 2021). The confidence intervals and *p*-values were corrected for multiple comparisons by using the Tukey method. Equation 1 depicts the general model in Wilkinson notation.

1 $$\textrm{Amplitude}\sim \textrm{Condition}\ast \textrm{Cue}\ast \textrm{PSWQ}\ast \textrm{MASQ}\ \textrm{AA}+\left(1\ |\ \textrm{Participant}\right)$$

## Results

### Startle

**Fig. 3:**
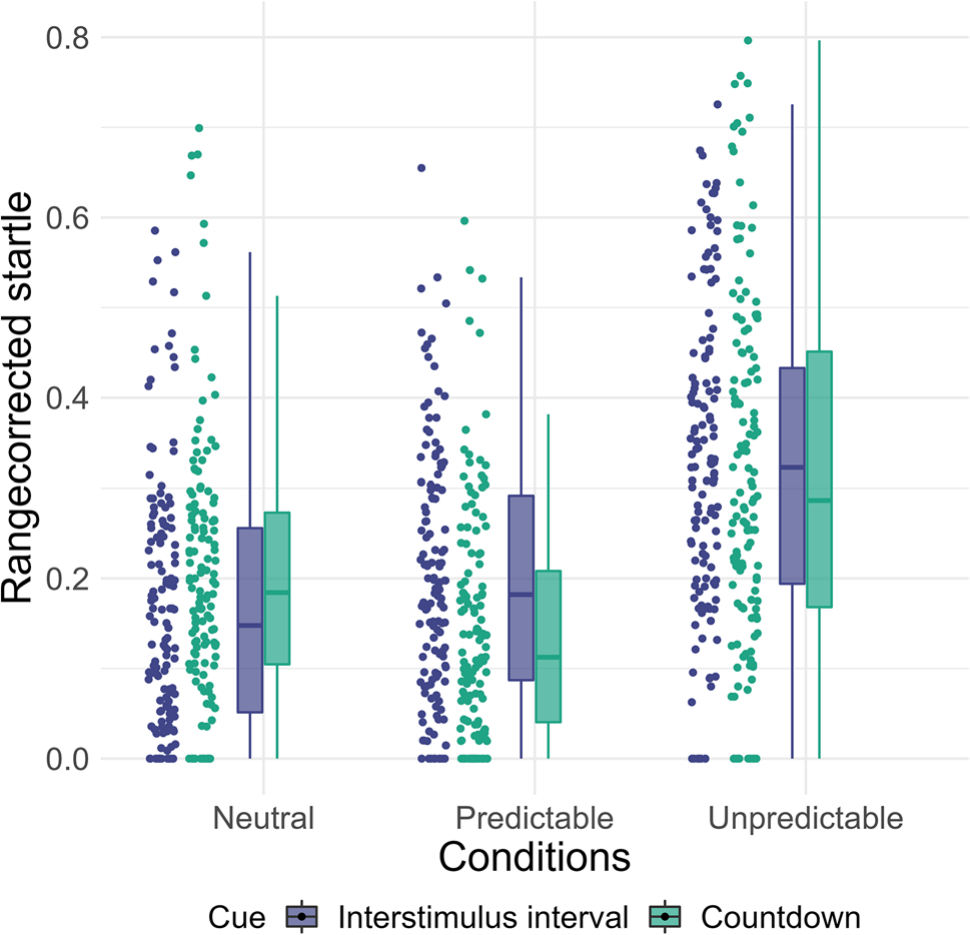
Participant’s mean startle responses across the conditions and cues. *Note*. Raw startle amplitudes were range corrected within participants to control for individual differences in absolute blink magnitude. Each amplitude was divided by the maximal amplitude of the participant, so that all amplitudes lie between 0 and 1

The statistical details of the results for startle responses are shown in Table 1 (for descriptive statistics see Supplement A). Startle responses in the unpredictable condition were increased compared with the neutral condition, whereas startle responses in the predictable condition were decreased compared with the neutral condition. In the unpredictable condition, participants showed increased startle responses to the countdown and interstimulus interval (Table 1). However, in the predictable condition, startle responses were smaller during the countdown compared with the interstimulus interval (Table 1; Fig. 3).

**Table 1:** Multilevel regression models predicting raw startle responses to the NPU-threat test

Fixed effects	*b*	*SE*	95% CI	*df*	*t*	*R*^*2*^
Intercept	32.36	4.03	24.46, 40.27	132	8.02	
Countdown	−0.63	0.59	−1.79, 0.52	4422	−1.07	.000
Predictable	−9.06	0.84	−10.70, −7.42	4422	−10.83	.026***
Unpredictable	16.15	0.93	14.52, 17.78	4422	19.37	.078***
PSWQ	0.26	0.32	−0.37, 0.88	132	0.81	.007
MASQ	−0.27	0.56	−1.36, 0.82	132	−0.49	.002
Countdown × Predictable	−2.44	0.84	−4.08, −0.80	4422	−2.92	.002**
Countdown × Unpredictable	−0.12	0.83	−1.76, 1.51	4422	−0.15	.000
Predictable × PSWQ	−0.15	0.07	−0.27, −0.02	4422	−2.21	.001*
Predictable × MASQ	−0.30	0.12	−0.52, −0.07	4422	−2.58	.001**
Unpredictable × PSWQ	0.36	0.07	0.26, 0.51	4422	5.90	.008***
Unpredictable × MASQ	0.25	0.11	0.03, 0.48	4422	2.22	.001*
PSWQ × MASQ	0.06	0.03	0.01, 0.12	132	2.15	.047*
Predictable × PSWQ × MASQ	0.00	0.00	−0.01, 0.01	4422	−0.13	.000
Unpredictable × PSWQ × MASQ	−0.01	0.01	−0.02, 0.01	4422	−1.08	.000
Random components						
σ^2^	1417.55					
τ_00_	1927.18					
ICC	.53					
Marginal *R*^2^	.069					
Conditional *R*^2^	.606					
*N*_participants_	136					
Observations	4578					

Cross-level interactions involving cue were not significant (*p*s > .211, *R*^2^s < .00). To reduce complexity, cross-level interactions involving cue are not shown here (but see Supplement B). For contrast coded conditions, neutral is the reference category, for contrast coded cues, interstimulus interval is the reference category. PSWQ = Penn-State Worry Questionnaire (grand-mean centered); MASQ = Mood and Anxiety Questionnaire (anxious arousal subscale, grand-mean centered); σ^2^ = residual variance; τ_00_ = random intercept; ICC = intra-class correlation;

***p < .001; **p < .01; *p < .05;

See Supplement E-F for analyses of the *t*-transformed and range corrected startle data

Concerning the anxiety dimensions, the analysis yielded condition × PSWQ interactions for predictable and unpredictable threat (Table 1). Follow-up contrasts of the slopes of PSWQ revealed that higher PSWQ predicted increased startle to unpredictable threat compared with both other conditions (unpredictable vs. predictable threat: *b* = 0.53, *SE* = 0.11, *t*(4422) = 4.68, *p* < .001; unpredictable threat vs. neutral condition: *b* = 0.62, *SE* = 0.11, *t*(4422) = 5.53, *p* < .001). The PSWQ slopes in the predictable condition did not differ significantly from the neutral condition (*b* = 0.09, *SE* = 0.11, *t*(4422) = 0.83, *p* = .682). A condition × MASQ-AA interaction (Table 1) indicates that participants with increased MASQ-AA scores showed increased startle in the unpredictable compared with the predictable condition (*b* = 0.55, *SE* = 0.20, *t*(4422) = 2.77, *p* = .016). No evidence emerged for differences between the slopes in the unpredictable and neutral condition (*b* = 0.21, *SE* = 0.20, *t*(4422) = 1.07, *p* = .535) or the predictable and neutral condition respectively (*b* = −0.34, *SE* = 0.20, *t*(4422) = −1.71, *p* = .201). The PSWQ × MASQ-AA interaction (Table 1) entailed contrast of the MASQ-AA slopes centered at ± 1 *SD* of PSWQ. Participants with higher PSWQ and higher MASQ-AA showed increased overall startle compared with participants with lower PSWQ and higher MASQ-AA (*b* = 1.70, *SE* = 0.79, *t*(132) = 2.15, *p* = .033). In other words, overall startle was increased in participants with increased scores in both anxiety dimensions. The fixed effects in the model explained 6.90% of the variance in the data, combined with the random effects of the model, 60.60% of the variance of the data were explained. To test the robustness of the effects against the pre-processing choice to use raw-startle magnitude in multilevel models (Carsten et al., 2022; Faunce et al., 2022), the analyses were repeated with *t*-transformed (Supplement E) and range corrected (Supplement F) startle amplitudes.^1^

### Event-related potentials

#### N1

The statistical details of the results for startle probe locked N1 are shown in Table 2. The probe locked N1 was increased (i.e., more negative) during unpredictable threat anticipation, compared with the neutral condition. In contrast, the probe locked N1 was decreased (i.e., less negative) during predictable threat anticipation, compared with the neutral condition (Table 2; Fig. 5). No evidence emerged for differences between the cues (i.e., countdown, interstimulus interval), and no condition × cue interaction emerged (Table 2).

**Table 2:** Multilevel regression models predicting probe locked N1 to the NPU-threat test

Fixed effects	*b*	*SE*	95% CI	*df*	*t*	*R*^*2*^
Intercept	−10.75	0.53	−11.79, −9.71	790	−20.23	
Countdown	0.04	0.12	−0.20, −0.28	790	0.30	.000
Predictable	1.65	0.17	1.31, 1.99	790	9.53	.066***
Unpredictable	−2.18	0.17	−2.52, −1.84	790	−12.56	.109***
PSWQ	0.03	0.04	−0.06, 0.11	790	0.64	.006
MASQ	−0.03	0.07	−0.17, 0.11	790	−0.41	.002
Countdown × Predictable	0.27	0.17	−0.07, 0.61	790	1.56	.002
Countdown × Unpredictable	−0.05	0.17	−0.39, 0.29	790	−0.32	.000
Predictable × PSWQ	0.01	0.01	−0.02, 0.04	790	0.81	.001
Predictable × MASQ	0.06	0.02	0.02, 0.11	790	2.63	.005*
Unpredictable × PSWQ	−0.01	0.01	−0.04, 0.01	790	−0.92	.001
Unpredictable × MASQ	−0.05	0.02	−0.10, −0.01	790	−2.21	.004*
PSWQ × MASQ	−0.01	0.00	−0.02, −0.00	790	−2.02	.056*
Predictable × PSWQ × MASQ	0.00	0.00	−0.00, 0.00	790	0.28	.000
Unpredictable × PSWQ × MASQ	0.00	0.00	−0.00, 0.00	790	0.38	.000
Random components						
σ^2^	10.93					
τ_00_	32.38					
ICC	.70					
Marginal *R*^2^	.092					
Conditional *R*^2^	.771					
*N*_participants_	136					
Observations	816					

Cross-level interactions involving cue are not shown (see Supplement C). For contrast coded conditions, neutral is the reference category, for contrast coded cues, interstimulus interval is the reference category. PSWQ = Penn-State Worry Questionnaire (grand-mean centered); MASQ = Mood and Anxiety Questionnaire (Anxious Arousal subscale, grand-mean centered); σ^2^ = residual variance; τ_00_ = random intercept; ICC = Intra class correlation;

***p < .001; **p < .01; *p < .05

Regarding the anxiety dimensions, significant interactions between the threat conditions and MASQ-AA emerged, as well as a MASQ-AA × PSWQ interaction (Table 2). Follow-up contrasts of the slopes of MASQ-AA revealed that increased MASQ-AA values led to increased N1 in the unpredictable condition compared with the predictable condition (*b* = −0.12, *SE* = 0.04, *t*(660) = −2.80, *p* = .015), whereas no evidence emerged for differences between the slopes of the unpredictable vs. neutral (*b* = −0.04, *SE* = 0.04, *t*(660) = −1.03, *p* = .559) or predictable vs. neutral conditions (*b* = 0.07, *SE* = 0.04, *t*(660) = 1.77, *p* = .181). The PSWQ × MASQ-AA interaction (Table 2) was followed by contrasts of the MASQ-AA slopes centered at ±1 *SD* of PSWQ. Overall, participants with higher PSWQ and higher MASQ-AA showed increased (i.e., more negative) N1 compared with participants with lower PSWQ and higher MASQ-AA (*b* = −0.21, *SE* = 0.10, *t*(132) = −2.02, *p* = .046). Thus, overall N1 was increased in participants with increased scores in both anxiety dimensions. An ICC of 0.70 indicates small variability within and large variability between participants. Combined, the fixed and random effects in the model explained 77.10% of the variance in the N1 data, whereas the fixed effects explained 9.20% of the variance.

#### P3

The statistical details of the results for probe locked P3 are shown in Table 3. Note that increased (i.e., more positive) P3 could be interpreted as increased allocation of attentional resources to the startle probe and not the task-associated threat stimuli (Nelson, Hajcak, et al., 2015a). During predictable threat anticipation, the probe locked P3 was decreased compared with the neutral condition (Table 3; Fig. 6). During unpredictable threat anticipation, the P3 did not differ significantly from the neutral condition. In the predictable threat condition, the participants showed increased probe locked P3 during the countdown compared with the interstimulus interval (Table 3; contrast of the estimated marginal means: *b* = 1.34, *SE* = 0.41, *t*(660) = 3.29, *p* = .013). In the neutral condition the P3 during the countdown and interstimulus interval did not differ significantly (*b* = 0.45, *SE* = 0.41, *t*(660) = 1.10, *p* = .881).

**Table 3:** Multilevel regression models predicting probe locked P3 to the NPU-threat test

Fixed effects	*b*	*SE*	95% CI	*df*	*t*	*R*^*2*^
Intercept	4.75	0.33	4.10, 5.39	790	14.36	
Countdown	0.29	0.12	0.06, 0.52	790	2.48	.006*
Predictable	−0.98	0.17	−1.31, −0.66	790	−5.91	.030***
Unpredictable	0.07	0.17	−0.26, 0.39	790	0.41	.000
PSWQ	0.02	0.03	−0.03, 0.07	790	0.76	.004
MASQ	−0.06	0.05	−0.15, 0.03	790	−1.40	.014
Countdown × Predictable	0.38	0.17	0.05, 0.70	790	2.28	.005*
Countdown × Unpredictable	−0.31	0.17	−0.64, 0.02	790	−1.87	.003
Predictable × PSWQ	0.03	0.01	0.00, 0.05	790	2.03	.004*
Predictable × MASQ	−0.01	0.02	−0.06, 0.03	790	−0.54	.000
Unpredictable × PSWQ	−0.01	0.01	−0.03, 0.02	790	-0.66	.000
Unpredictable × MASQ	−0.02	0.02	−0.06, 0.03	790	-0.82	.001
PSWQ × MASQ	−0.00	0.00	−0.01, 0.00	790	-0.98	.007
Predictable × PSWQ × MASQ	0.00	0.00	−0.00, 0.00	790	1.12	.001
Unpredictable × PSWQ × MASQ	0.00	0.00	−0.00, 0.00	790	0.22	.000
Random components						
σ^2^	10.02					
τ_00_	11.57					
ICC	.52					
Marginal *R*^2^	.061					
Conditional *R*^2^	.564					
*N*_participants_	136					
Observations	816					

Cross-level interactions involving cue are not shown here (see Supplement D). For contrast coded conditions, neutral is the reference category, for contrast coded cues, interstimulus interval is the reference category. PSWQ = Penn-State Worry Questionnaire (grand-mean centered); MASQ = Mood and Anxiety Questionnaire (anxious arousal subscale, grand-mean centered); σ^2^ = residual variance; τ_00_ = random intercept; ICC = Intraclass correlation; ***p < .001; **p < .01; *p < .05

Concerning the anxiety dimensions, a significant cross-level interaction between the predictable condition × PSWQ emerged (Table 3), which stems from increased P3 in participants with higher PSWQ in the predictable compared with the neutral condition (contrast of the slopes of PSWQ: *b* = 0.05, *SE* = 0.02, *t*(660) = 1.96, *p* = .122; Figure 7B). Notably, the significant interaction in the effect-coded model (Table 3) did not survive the Tukey correction of the post-hoc tests. No evidence emerged for differences of the PSWQ slopes in the unpredictable and neutral condition (*b* = 0.01, *SE* = 0.02, *t*(660) = 0.41, *p* = .912) or predictable and unpredictable condition (*b* = 0.04, *SE* = 0.03, *t*(660) = 1.56, *p* = .489). PSWQ and MASQ-AA did not interact in predicting the P3 (Table 3). An ICC of .52 indicates large variability between participants. The fixed and random effects of the model combined accounted for 56.40% of the variance in the data.

## Discussion

Anxiety maps onto the symptom dimensions anxious apprehension and anxious arousal, but the underlying neural mechanisms are unclear. This study was designed to examine whether individual differences in worry—as a key component of anxious apprehension—and anxious arousal predict state changes in the neural processing of aversive stimuli. Participants were recruited from a large, online, community sample to oversample individuals with converging and diverging profiles on the anxiety dimensions. This approach was taken to examine possible interactions between worry and anxious arousal that might modulate the dynamics of threat processing. Outcome measures included the startle reflex and startle probe locked ERP components N1 and P3. In line with previous studies, we found increased startle to unpredictable threat (Carsten et al., 2022; Ferry and Nelson, 2020; Gorka et al., 2017; Nelson et al., 2016), which was simultaneously mirrored in enhanced probe locked N1 reflecting increased early sensory processing. The probe locked P3 was decreased during predictable threat anticipation reflecting reduced attention allocation to the startle probe during the anticipation of predictable threat. Regarding the anxiety dimensions, we found three main results. First, worry was associated with increased startle to unpredictable threat. Second, worry was linked to increased probe locked P3 during predictable threat. Third, anxious arousal was associated with increased startle and probe locked N1 during unpredictable threat compared with predictable threat. Together, these findings imply that individual differences in worry and anxious arousal alter the psychophysiological response patterns to threat anticipation.

### Startle

Startle responses during temporally unpredictable threat anticipation were increased compared with the neutral condition, which is in line with task effects observed in previous studies (Nelson and Shankman, 2011; Schmitz and Grillon, 2012). During the anticipation of predictable threat, startle responses are usually increased (Bradford et al., 2014; Gorka, Nelson, and Shankman, 2013; Schmitz and Grillon, 2012). Contrary to our expectations, we found reduced startle responses during the countdown in the predictable compared with the neutral and unpredictable threat condition. This counterintuitive finding may indicate that individuals display decreased defensive preparedness in anticipation of temporally predictable and imminent aversive stimuli. But our finding could also be due to a combination and/or interaction of experimental factors, such as the choice of an auditory aversive stimulus, relatively low startle probe volume, and a visual countdown as the cue stimulus. Specifically, we used an auditory aversive stimulus, which might explain why startle responses during the presentation of the countdown in the predictable condition were reduced. Ferry and Nelson (2020) also found decreased startle in anticipation of predictable screams but not in anticipation of shocks. Thus, fear potentiated startle in the NPU-threat test might depend on the modality of the aversive stimulus. The inherent social component of the auditory scream might have triggered a response preparation, which has been shown to link to decreased startle (Löw et al., 2015). Another possible explanation consists in the comparably low volume of the startle probes. Here, the volume of startle probes was 93 dB. Other studies report volumes ranging from 95 dB (Carsten et al., 2022; Nelson and Shankman, 2011) to 103 dB (Ferry and Nelson, 2020; Gorka et al., 2013, 2017; Grillon et al., 2017; Lieberman, Gorka, et al., 2017a; Lieberman, Stevens, et al., 2017b; Morriss, Biagi, and Dodd, 2020a; Nelson et al., 2016; Nelson, Hajcak, et al., 2015a; Nelson, Hodges, et al., 2015b; Shankman et al., 2013). This may have intensified the difference in salience between the startle probe and aversive stimulus (100 dB) in our study. Together with a precisely predictable aversive stimulus in the predictable threat condition due to the use of a countdown as the preceding threat cue (as opposed to geometric shapes; MacNamara and Barley, 2018; Schmitz and Grillon, 2012; Shankman et al., 2013), this salience difference might have enabled the participants to block out the acoustic startle probes in the predictable condition compared with the unpredictable threat condition, in which startle responses were increased. In summary, reduced startle in anticipation of predictable threat might either point to decreased defensive preparation during temporally predictable threat or result from a high degree of threat predictability and threat imminence. Future studies could elucidate this question by extending previous findings that disentangle effects of variations in the experimental setup of the NPU-threat test (Ferry and Nelson, 2020).

**Fig. 4:**
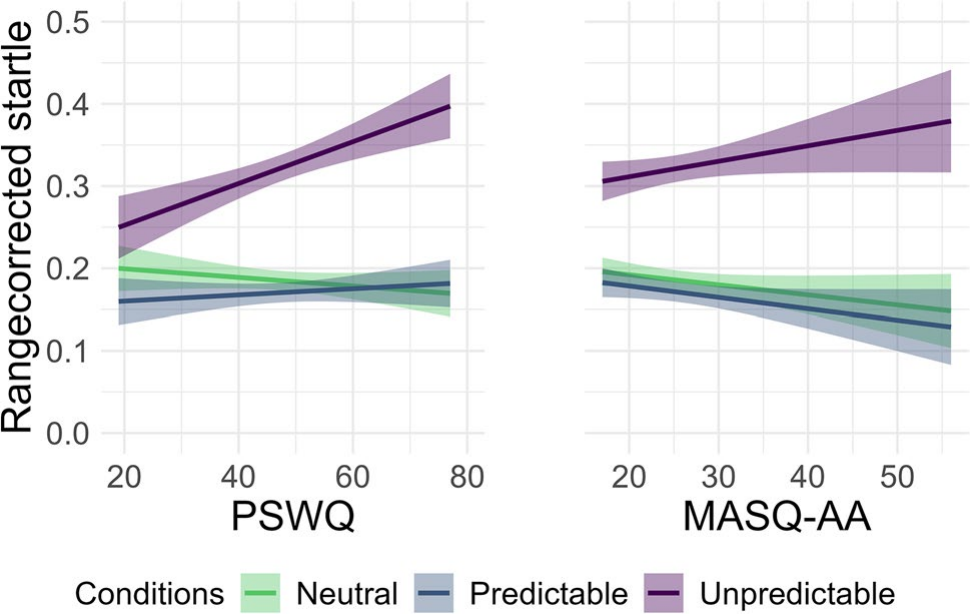
Startle slopes of the Penn-State Worry Questionnaire (PSWQ) and the anxious arousal subscale of the Mood and Anxiety Symptom Questionnaire (MASQ-AA) across the experimental conditions of the NPU-threat test. *Note*. Data are collapsed across cues (interstimulus interval, countdown), because no modulation by cue was observed. The shaded area indicates a 95% confidence interval of the estimated slope. Raw startle amplitudes were range corrected within participants to control for individual differences in absolute blink magnitude. Each amplitude was divided by the maximal amplitude of the participants, so that all amplitudes lie between 0 and 1

Worry, as measured by the PSWQ, exerted a modulating effect on startle responses in anticipation of unpredictable threat: Increased PSWQ values led to increased startle during unpredictable threat anticipation compared with the neutral condition (Figure 4). This adds to previous but inconsistent studies investigating the role of worry and generalized anxiety disorder in the neural processing of threat (Gorka et al., 2017; Grillon et al., 2009, 2017; Nelson and Shankman, 2011; Rutherford et al., 2020). As such, our findings suggest that in subclinical individuals greater worry is associated with increased automatic defensive preparedness in unpredictable threatening contexts. Individuals who are hypersensitive to unpredictable contexts might habitually have adopted worrying to decrease the uncertainty arousal associated with unpredictable situations (Einstein, 2014; Sibrava and Borkovec, 2006). However, this finding is in contrast to results of Nelson and Shankman (2011), who found no evidence for PSWQ modulating startle in the NPU-threat test in a sample of introductory psychology students. Similarly, our findings are contrary to Rutherford et al. (2020), who even found decreased startle to unpredictable threat in participants with higher PSWQ values, but only if they had a history of anxiety disorders. Importantly, Rutherford et al. (2020) manipulated the occurrence probability of threat, whereas in the present study, the temporal predictability was manipulated. Previous studies suggest that associations between anxiety-related traits and threat anticipation might be sensitive to specifics aspects of predictability. As such, individual differences in intolerance of uncertainty (IU; Carleton, 2016a, 2016b) increased defensive responding to threat with unpredictable or lowered occurrence probability (Carsten et al., 2022; Chin et al., 2016, but see Bennett et al., 2018).

**Fig. 5:**
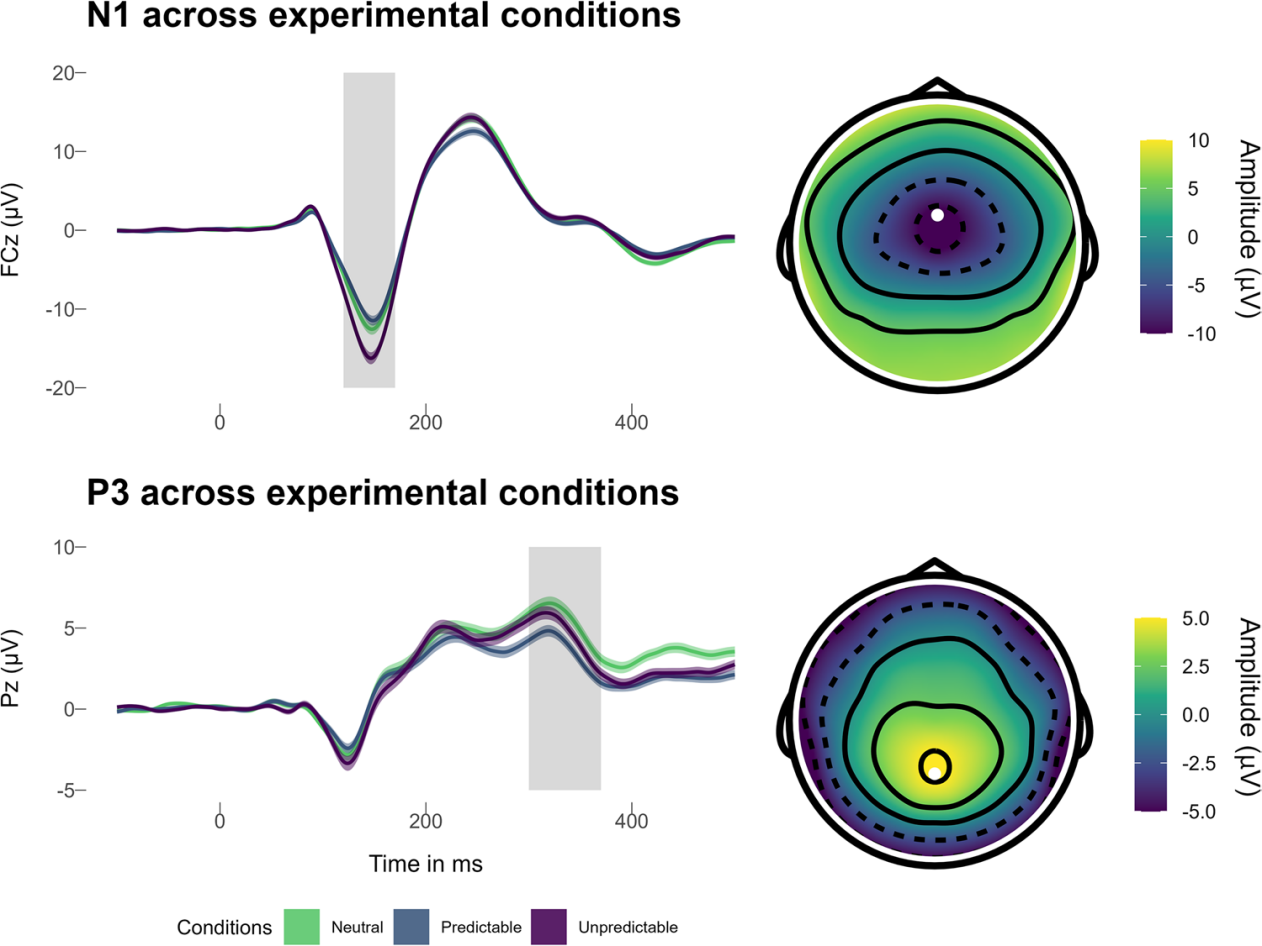
Grand averages across experimental conditions (left) and head maps (right) of the event related potentials N1 (top) and P3 (bottom). *Note*. The signal is locked to startle probes and is collapsed for the grand averages (left) for illustration across cues of the NPU-threat test. The time windows for quantification of N1 (±25 ms area around the individual peak at FCz; white dot; ca. 120-170 ms) and P3 (mean activity 300–370 ms at Pz; white dot) are indicated by shaded grey bars. The shaded area around the mean signal depicts ± standard error of the distribution at each ms. The head maps (right) show the grand average signal collapsed across the conditions and cues of the NPU-Threat test

**Fig. 6:**
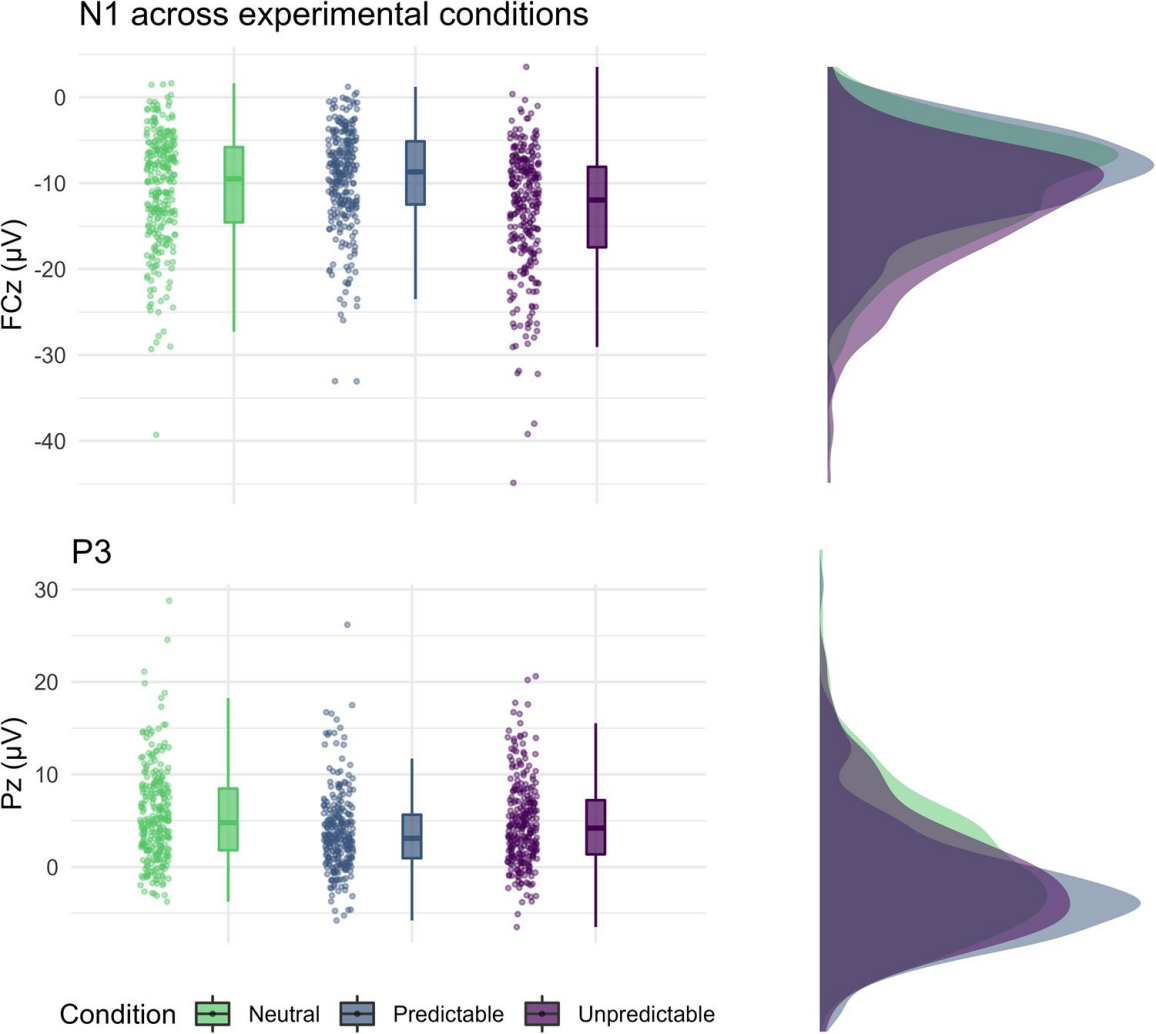
Jitter plot, box plot and density plots of the N1 (top) and P3 (bottom) across the conditions of the NPU-threat test. *Note.* Data are collapsed across the cues (countdown and interstimulus interval)

**Fig. 7:**
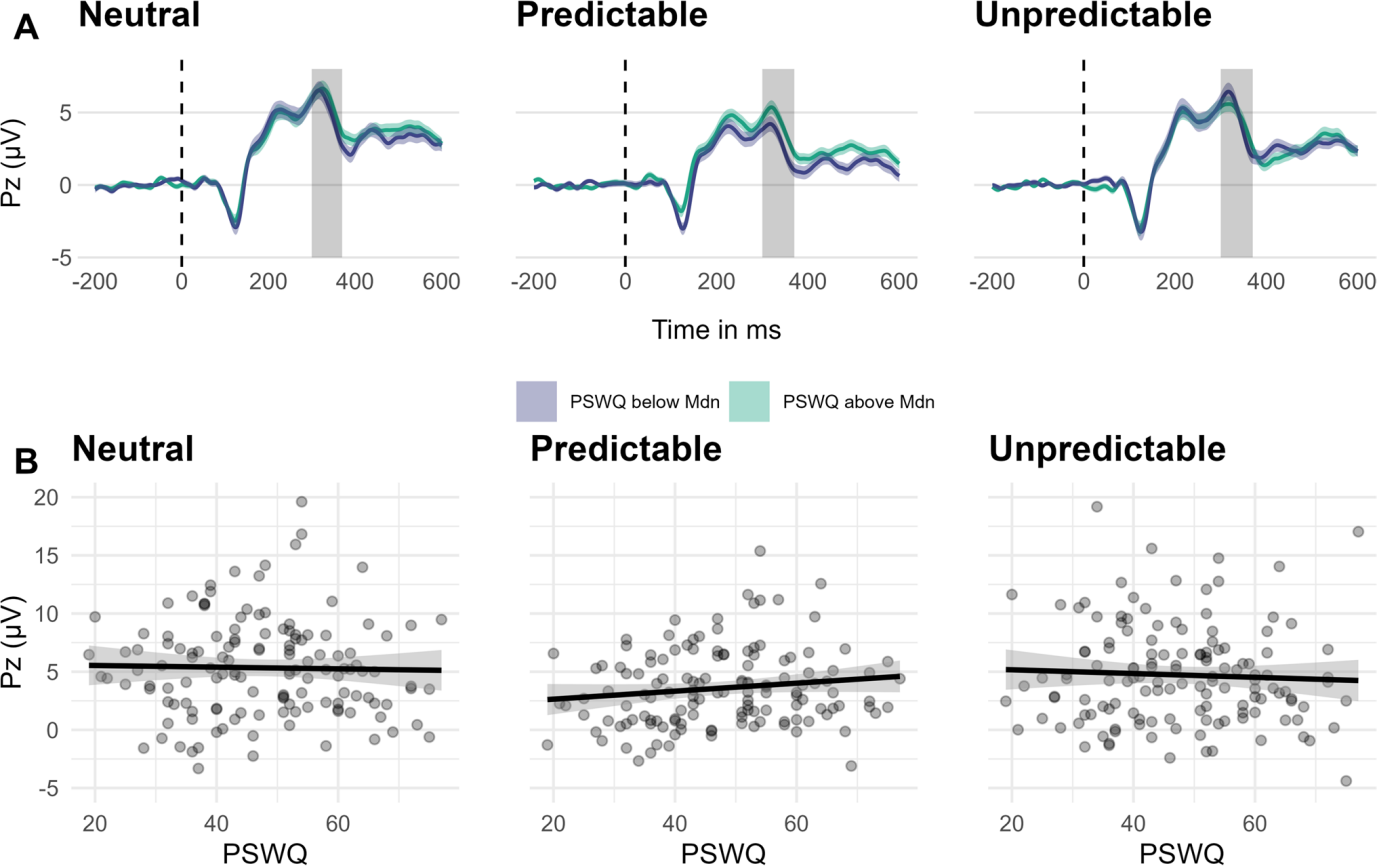
Averages (**A**) and slopes (**B**) per condition of the NPU-threat test by PSWQ. *Note.* **A.** Averages of P3 across the conditions of the NPU-threat test for participants below and above the median split (47, color-coded) of the Penn-State Worry Questionnaire (PSWQ). Shaded areas around the signal depict the standard error at each ms; the shaded grey areas show the time window for quantification of the P3 (mean activity 300–370 ms). The dashed line indicates the onset of the startle probe. **B.** Slopes of the PSWQ and P3 across the experimental conditions of the NPU-threat test. The shaded area around the slope depicts the 95% confidence interval of the slope estimate. Data are collapsed across cues (countdown, interstimulus interval)

Together, a role of worry in defensive responding to unpredictable threat is likely. Yet, the direction of effects warrants further investigation, because there is inconsistency between studies finding decreased defensive responding to unpredictable threat (Rutherford et al., 2020) and increased anxiety potentiation (indicated by the present findings). This might help to integrate the mixed prior findings of clinical studies on generalized anxiety disorder (Gorka et al., 2017; Grillon et al., 2009, 2017). One possible explanation for the observed inconsistencies could be that patients with an anxiety disorder and/or with increased worry might display concurring overall attenuated physiological responses and an increased anxiety potentiation. In a clinical population, Lang et al. (2016) found evidence for decreased physiological responding as a function of distress/broad negative affectivity, the generalization of anxiety and functional impairment. Our results suggest that worry as a specific symptom dimension often accompanying negative affectivity might actually increase defensive responding to unpredictable threat in the subclinical spectrum of anxiety. Controlling for negative affect by means of BDI-II did not change this pattern of results, suggesting that it might be more specific to worry. Elevated levels of worry are present in more generalized anxiety with generalized anxiety disorder as the clinical phenotype and prototype (Cox et al., 2010; Krueger, 1999). Thus, future studies should focus on factors such as clinical status, functional impairment, and comorbidity, to broaden our understanding of the isolated effect of worry on unpredictable threat anticipation. Another avenue for future research is to extend the focus from anxiety-related traits to more fear-related traits (Corr, 2009) to understand how trait-fearfulness might translate to physiological response patterns (Panitz et al., 2018). This might help to understand which transdiagnostic symptom dimensions shape responses to threat in more focal fears (e.g., specific phobia) versus more generalized anxiety (e.g., generalized anxiety disorder).

As a more fear-related construct, in the current study, anxious arousal was associated with increased startle responses in unpredictable threat anticipation relative to predictable threat anticipation (as indicated by an Unpredictable × MASQ interaction). Hence, individuals, who report increased anxious arousal, might show increased defensive preparedness in unpredictable aversive contexts. This could be more specific to unpredictability than to the presence of threat. A similar pattern of results related to anxious arousal emerged in startle probe locked N1.

### N1

Regarding general task effects and in line with previous findings, startle probe locked N1 was increased (i.e., more negative) during unpredictable threat anticipation (Ferry and Nelson, 2020; Nelson, Hajcak, et al., 2015a; Nelson and Hajcak, 2017), which mirrors the effect observed in startle. In addition, we found a decreased auditory processing of startle probes during predictable threat anticipation as indicated by a reduced N1 in the predictable threat condition relative to the neutral condition. This finding converges with reduced startle responses during the predictable condition. Together, reduced startle and N1 during the predictable threat condition point to decreased defensive preparation during temporally predictable threat and could result from a high degree of threat predictability and threat imminence in our task.

Anxious arousal was associated with increased (i.e., more negative) probe locked N1 in unpredictable threat anticipation relative to predictable threat anticipation. In essence, MASQ-AA predicting both—increased startle and N1—in the unpredictable condition compared with the predictable condition could indicate that individuals with increased trait anxious arousal respond automatically to unpredictable aversive contexts. This conveys preliminary evidence for a signature of hyperreactivity to unpredictable threat across psychophysiological units of analysis in individuals with increased anxious arousal. Although this effect emerged for startle and N1 in conjunction, it should be interpreted as exploratory. There are no previous data or clear theoretical predictions pointing to this specific pattern of results, and the effect sizes were small (startle: *R*^2^ = .001; N1: *R*^2^ = .004). For startle, the effect was sensitive to pre-processing choices and was statistically significant for raw and range corrected startle but not for *t*-transformed startle. Furthermore, the MASQ-AA slopes in startle and N1 differed significantly only between unpredictable and predictable conditions, but not between the unpredictable and neutral condition. Future studies could try to replicate this effect and test whether it holds true or is actually increased in clinical populations.

### P3

In line with previous studies, the startle probe locked P3 was decreased during predictable threat anticipation suggesting decreased attentional allocation (i.e., orienting response) to task-irrelevant threat stimuli (i.e., startle probe) when a threat is temporally predictable and imminent (Ferry and Nelson, 2020; MacNamara and Barley, 2018; Nelson, Hajcak, et al., 2015a; Nelson and Hajcak, 2017). Unexpectedly, the probe locked P3 in predictable threat anticipation was more decreased during the interstimulus interval compared with the countdown, indicating that this preparatory focus of attention on predictable threatening stimuli (i.e., blunted probe locked P3) might already be initiated by the instruction during the interstimulus interval.

Concerning the anxiety dimensions, we found evidence for an effect of worry on the attentional allocation to the startle probe during the anticipation of predictable threat: Increased PSWQ scores were associated with an increased P3 in the predictable condition. Previous research found decreased probe locked P3 during the anticipation of (predictable) threat (Ferry and Nelson, 2020; MacNamara and Barley, 2018; Nelson, Hajcak, et al., 2015a). This is considered to indicate that attentional resources are directed to the threat cue rather than the task-irrelevant startle probe, which might be an adaptive strategy in the face of predictable threat. Our results suggest that individuals with increased trait worry seem to have difficulty in this preparatory focus of attention (Forster et al., 2015; Morriss, Biagi, and van Reekum, 2020b). This indicates a decreased ability to distinguish between predictable and unpredictable situations in individuals with elevated worry, which could render predictable situations more uncertain and thus aversive. This attentional bias might result in a quantitative difference in experiences of uncertainty and could thereby contribute to the development and maintenance of anxiety and intolerance of uncertainty (Carleton, 2016b). But the reverse path also is possible, wherein anxious apprehension and worrying are conceptualized as a coping mechanism to reduce uncertainty (i.e., a consequence of intolerance of uncertainty; Einstein, 2014). The absence of evidence for PSWQ modulating earlier neural processing (N1) during the anticipation of predictable threat could be taken to suggest that worry mitigates threat responses in a predictable context primarily by means of attentional allocation.

Overall, our findings suggest that worry increases defensive responding in anticipation of unpredictable threat (as indexed by increased startle in the unpredictable condition) and increases attentional allocation to irrelevant stimuli during predictable threat anticipation (as indexed by increased P3 to startle probes in the predictable condition in participants with increased worry). Anxious arousal might be associated with increased defensive responding to unpredictable threat relative to predictable threat.

### Limitations

Several limitations should be considered when interpreting the present findings. First, findings on predictable threat anticipation should be interpreted with caution, because the typical potentiation for predictable threat anticipation did not emerge in startle or N1. However, an expected task effect regarding predictable threat anticipation was evident in probe locked P3, which was decreased compared with the the neutral condition. Future studies might build on these findings and examine boundary effects of the NPU-threat test, e.g., by comparing the sensory modality (Ferry and Nelson, 2020) or intensity of the aversive stimuli. Along these lines, it might be helpful to remove sources of potential threat during the neutral condition, i.e., to exclude potentially aversive startle probes that might render the neutral condition unsafe. Second, it remains unclear whether the role of the anxiety dimensions might be increased or different in clinical manifestations of anxiety symptomatology. Thus, the results of the present study allow inferences about trait worry, but the link to clinical manifestations of anxiety and hence inferences relating to the generalization of anxiety (Lang et al., 2016) remain indirect. Although participants were not screened for lifetime anxiety disorders, an inclusion of participants with prior or current anxiety disorders—but intact functional adaptability—is likely, because we oversampled participants with diverging anxiety dimension profiles and anxiety disorders are extensively prevalent in the general population (Baxter et al., 2013). Previous findings could be taken to suggest categorical differences between individuals with a lifetime anxiety disorder versus without (Rutherford et al., 2020). In the current study, we did not screen for current or prior anxiety disorders, which makes it impossible to identify a clinical threshold for the effects of interest. Instead, we followed the central ideas of the RDoC approach (Insel et al., 2010), proposing a continuum for psychopathology with close relations to underlying neurophysiological systems. In this vein, our dimensional analytic strategy conveyed no evidence for a bimodal distribution of startle to unpredictable threat anticipation that would suggest a clinical threshold of the effects (Sharp et al., 2015).

Third, no data were collected on ethnicity or nationality, which limits the interpretation of the generalizability of our results. However, the online screening was made available to the general population in Berlin, of which we included participants based on their trait worry and anxious arousal profiles. The participant’s age ranged from 18 to 62 years (*M* = 28.60, *SD* = 9.22), with *M* = 13.30 (*SD* = 2.06) years of education. Thus, although data on ethnicity and nationality is missing, we recruited a community sample, which ensures a higher degree of generalizability compared with the frequently used samples of undergraduates. By operationalizing anxious apprehension by means of the PSWQ, we focused on a specific symptom dimension (i.e., worry) that characterizes anxious apprehension. Further aspects of anxious apprehension such as beliefs related to uncertainty and uncontrollability, i.e., intolerance of uncertainty, might further play a role in attentional and defensive responding to unpredictable threats (Carsten et al., 2022; Correa et al., 2022; Morriss, Biagi, and Dodd, 2020a). Lastly, the PSWQ and MASQ-AA were not readministered during the laboratory assessment. Consequently, we cannot rule out a change in trait levels of anxious apprehension and anxious arousal from the online screening to the laboratory assessment.

Next to these limitations, the present study comprises considerable strengths. Due to a comparably large sample size, the statistical analyses were powered to detect even small effects. This is especially true for the startle data, as no data aggregation, as compared with more traditional ANOVA approaches, was necessary. Moreover, employing MLMs enabled us to model individual differences in physiological response patterns with more granularity by accounting for the dimensional data structure of the questionnaires. Across outcome measures, the data exhibited significant clustering (as indicated by ICCs), which confirms the inclusion of random effects. Using these granular analytic approaches, we aimed at disentangling possible interaction effects of worry and anxious arousal specific to threat processing, which were not evident in the data. This further corroborates the separability of the anxiety dimensions (Sharp et al., 2015) and indicates that trait levels of worry and anxious arousal differentially translate to state changes across different units of analysis or that the interrelations between trait levels and state changes are too small to be detectable in the current, comparably large sample.

### Conclusions

In consideration of these limitations and strengths, the present study conveys evidence for trait worry and anxious arousal as central anxiety dimensions that differentially modulate physiological and attentional aspects in threat processing. Particularly, trait variations of worry and anxious arousal allowed the prediction of state changes in psychophysiological responses to unpredictable threat anticipation. Participants with increased worry displayed increased startle in unpredictable threat anticipation compared with the predictable threat and neutral conditions. To a smaller degree, participants with increased anxious arousal displayed increased startle in unpredictable threat anticipation. Together, both anxiety dimensions explain unique variance in defensive preparedness to unpredictable aversive contexts. Individual differences in the anxiety dimensions might thus be reflected in altered physiological response patterns, such that individuals with elevated scores on either anxiety dimension—but more pronounced for worry—show amplified defensive preparedness in the face of uncertain threats. Furthermore, in a predictable, aversive context, worry might modulate the allocation of attentional resources to threat stimuli such that we observed a blunting in the preparatory focus of attention during predictable threat. Altogether, this suggests an essential role of trait worry in alterations of the sequential neural processing of threat stimuli and corroborates theoretical conceptualizations that highlight anxious apprehension as a central underlying symptom dimension in anxiety.

## Supplementary information

ESM 1 (PDF 601 kb)

## Footnotes

1 The effects of the NPU-threat test conditions converged across quantification approaches (i.e., increased startle for unpredictable threat and decreased startle during the predictable countdown). Increased startle in the unpredictable condition in participants with increased PSWQ (i.e., Unpredictable × PSWQ interaction, Table 1) also converged across quantifications. The Unpredictable × MASQ interaction only emerged for raw and range corrected startle. The Predictable × PSWQ, Predictable × MASQ, and PSWQ × MASQ interactions only emerged for raw startle.
