# Correction: Don’t worry, it won’t be fine. Contributions of worry and anxious arousal to startle responses and event-related potentials in threat anticipation

**DOI:** 10.3758/s13415-023-01109-0

**Published:** 2023-05-17

**Authors:** Hannes Per Carsten, Kai Härpfer, Brady D. Nelson, Norbert Kathmann, Anja Riesel

**Affiliations:** 1 Department of Psychology, University of Hamburg, Von-Melle-Park 11, 20146 Hamburg, Germany; 2 Department of Psychology, Stony Brook University, Stony Brook, NY USA; 3 Department of Psychology, Humboldt University of Berlin, Berlin, Germany

**Correction: Cognitive, Affective, & Behavioral Neuroscience**

10.3758/s13415-023-01094-4

The original article has been updated to correct the color coding of Figure 7A i.e., “PSWQ above Mdn” and “PSWQ below Mdn” was changed to “PSWQ below Mdn” and “PSWQ above Mdn”.

