# Supplementary material for: Don’t worry, it won’t be fine. Contributions of worry and anxious arousal to startle responses and event-related potentials in threat anticipation
Source: Cogn Affect Behav Neurosci. 2023 Apr 27;23(4):1141–59. doi: 10.3758/s13415-023-01094-4 (PMC10400686; doi:10.3758/s13415-023-01094-4)
Supplement: Supplementary file 1 — (PDF 601 kb) [file 13415_2023_1094_MOESM1_ESM.pdf]

Supplementary Material to

"Don't worry, it won't be fine. Contributions of worry and anxious arousal to startle responses and event-related potentials in threat anticipation."

Hannes Per Carsten[1]*, Kai Härpfer[1], Brady Nelson[2], Norbert Kathmann[3], & Anja Riesel[1]

[1]Department of Psychology, University of Hamburg, Hamburg, Germany

[2]Department of Psychology, Stony Brook University, NY, USA

[3]Department of Psychology, Humboldt University of Berlin, Berlin, Germany

*Correspondence concerning this article should be addressed to Hannes Per Carsten, Department of Psychology, Universität Hamburg, Von-Melle-Park 11, 20146 Hamburg, Germany.

E-Mail: hannes.carsten@uni-hamburg.de

Table of Contents:

Supplement A: Multilevel model for startle including interaction terms with cue 2

Supplement B: Multilevel model for raw startle including interaction terms with cue .. 3

Supplement C: Full multilevel model for N1 5

Supplement D: Full multilevel model for P3 7

Supplement E: *t*-transformed startle analyses 9

Supplement F: Range-corrected startle analyses 11

Supplement G: Power Simulations 13

Supplement H: Grand averages 15

Supplement A: Multilevel model for startle including interaction terms with cue

Table S1

		**Startle (μV)**	**Startle (*t*)**	**N1 (μV)**	**P3 (μV)**
**Condition**	**Cue**	*M* (*SD*)	*M* (*SD*)	*M* (*SD*)	*M* (*SD*)
Neutral	ISI	27.21 (45.83)	47.65 (7.41)	-10.39 (6.28)	5.23 (5.17)
	CD	31.07 (52.47)	49.01 (8.42)	-10.83 (6.97)	5.72 (4.93)
Predictable	ISI	29.44 (52.32)	48.79 (9.57)	-9.63 (5.74)	3.00 (3.79)
	CD	23.04 (40.68)	46.77 (7.06)	-9.23 (5.99)	4.44 (4.67)
Unpredictable	ISI	52.20 (75.36)	54.09 (11.89)	-13.38 (7.25)	4.74 (4.76)
	CD	50.94 (75.86)	53.66 (11.20)	-13.13 (7.45)	4.70 (4.59)

Note. ISI: Interstimulus interval. CD: Countdown

Supplement B: Multilevel model for raw startle including interaction terms with cue

Table S2

Multilevel model predicting range corrected startle responses to the NPU-Threat test. To calculate the range corrected amplitudes, each raw amplitude of a participant was divided by that participant's maximal amplitude and multiplied by 100 so that all amplitudes lie between 0 and 100.

**Fixed Effects**	***b***	***SE***	**95% *CI***	***df***	***t***	R^2
Intercept	32.36	4.03	24.45, 40.27	132	8.02	***
Countdown	-0.63	0.59	-1.79, 0.52	4424	-1.07	.000
Predictable	-9.06	0.84	-10.70, -7.42	4424	-10.83	.025***
Unpredictable	16.15	0.83	14.51, 17.78	4424	19.37	.076***
PSWQ	0.26	0.32	-0.37, 0.88	132	0.81	.002
MASQ	-0.27	0.55	-1.36, 0.82	132	-0.49	.003
Countdown × Predictable	-2.44	0.84	-4.08, -0.80	4424	-2.92	.004**
Countdown × Unpredictable	-0.12	0.83	-1.76, 1.51	4424	-0.15	.000
Countdown × PSWQ	-0.04	0.05	-0.13, 0.05	4424	-0.78	.000
Countdown × MASQ	0.04	0.08	-0.12, 0.20	4424	0.51	.000
Predictable × PSWQ	-0.15	0.07	-0.27, -0.02	4424	-2.21	.000*
Predictable × MASQ	-0.30	0.12	-0.52, -0.07	4424	-2.58	.001*
Unpredictable × PSWQ	0.39	0.07	0.26, 0.51	4424	5.90	.003***
Unpredictable × MASQ	0.25	0.11	0.03, 0.48	4424	2.22	.001*
PSWQ × MASQ	0.06	0.03	0.01, 0.12	132	2.15	.006*
Predictable × PSWQ × MASQ	-0.00	0.01	-0.01, 0.01	4424	-0.13	.000
Unpredictable × PSWQ × MASQ	-0.08	0.11	-0.30, 0.14	4424	-0.69	.000
Countdown × Predictable × PSWQ	-0.01	0.07	-0.13, 0.12	4424	-0.09	.000
Countdown × Predictable × MASQ	-0.06	0.12	-0.29, 0.16	4424	-0.53	.000
Countdown × PSWQ × MASQ	0.00	0.00	-0.01, 0.01	4424	0.33	.000
Countdown × Unpredictable × PSWQ	0.07	0.07	-0.06, 0.20	4424	1.07	.001
Countdown × Unpredictable × MASQ	-0.08	0.11	-0.30, 0.14	4424	-0.69	.000
Countdown × Predictable × PSWQ × MASQ	-0.01	0.01	-0.02, 0.00	4424	-1.25	.000
Countdown × Unpredictable × PSWQ × MASQ	0.01	0.01	-0.00, 0.02	4424	1.31	.000
**Random Components**						

σ^2	1417.55
τ_{00}	1928.18
ICC	.58
Marginal R^2	.069
Conditional R^2	.606
$N_{participants}$	136
Observations	4578

Note. For contrast coded conditions, neutral is the reference category, for contrast coded cues, interstimulus interval is the reference category. PSWQ = Penn-State Worry Questionnaire (grand-mean centered); MASQ = Mood and Anxiety Questionnaire (Anxious Arousal subscale, grand-mean centered); σ^2 = residual variance; τ_{00} = random intercept; ICC = Intra class correlation;
*** $p < .001$; ** $p < .01$; * $p < .05$

To test the specificity of the effects to PSWQ, we exploratorily included BDI-II into the model, which did not change the pattern of results regarding PSWQ. Specifically, the Unpredictable × PSWQ interaction remained significant ($b = 0.40$, $SE = 0.08$, $t(4422) = 5.14$, $R^2 = .006$). While the Predictable × MASQ interaction remained significant ($b = -0.27$, $SE = 0.13$, $t(4422) = -2.05$, $R^2 = .001$), the Unpredictable × MASQ interaction was no longer significant when including BDI-II into the model ($b = 0.18$, $SE = 0.13$, $t(4422) = 1.37$, $R^2 = .000$).

Supplement C: Full multilevel model for N1

Table S3

Multilevel model predicting startle probe locked N1 in µV to the NPU-Threat test.

**Fixed Effects**	***b***	***SE***	**95% *CI***	***df***	***t***	***R***^{2}
Intercept	-10.75	0.53	-11.79, -9.71	132	-20.23	***
Countdown	0.04	0.12	-0.20, 0.28	660	0.30	.000
Predictable	1.65	0.17	1.31, 1.99	660	9.53	. 066***
Unpredictable	-2.18	0.17	-2.52, -1.84	660	-12.56	.109***
PSWQ	0.03	0.04	-0.06, 0.11	132	0.64	.006
MASQ	-0.03	0.07	-0.17, 0.11	132	-0.41	.002
Countdown × Predictable	0.27	0.17	-0.07, 0.61	660	1.56	.002
Countdown × Unpredictable	-0.05	0.17	-0.39, 0.29	660	-0.32	.000
Countdown × PSWQ	0.01	0.01	-0.01, 0.02	660	0.58	.000
Countdown × MASQ	-0.00	0.02	-0.03, 0.03	660	-0.10	.000
Predictable × PSWQ	0.01	0.01	-0.02, 0.04	660	0.81	.001
Predictable × MASQ	0.06	0.02	0.02, 0.11	660	2.63	.006**
Unpredictable × PSWQ	-0.01	0.01	-0.04, 0.01	660	-0.92	.001
Unpredictable × MASQ	-0.05	0.02	-0.10, -0.01	660	-2.21	.004*
PSWQ × MASQ	-0.01	0.00	-0.02, -0.00	132	-2.02	.056*
Predictable × PSWQ × MASQ	0.00	0.00	-0.00, 0.00	660	0.28	.000
Unpredictable × PSWQ × MASQ	0.00	0.00	-0.00, 0.00	660	0.38	.000
Countdown × Predictable × PSWQ	0.02	0.01	-0.01, 0.05	660	1.38	.001
Countdown × Predictable × MASQ	0.04	0.02	-0.01, 0.09	660	1.74	.002
Countdown × PSWQ × MASQ	-0.00	0.00	-0.00, 0.00	660	-0.07	.000
Countdown × Unpredictable × PSWQ	-0.00	0.01	-0.03, 0.02	660	-0.16	.000
Countdown × Unpredictable × MASQ	-0.03	0.02	-0.08, 0.02	660	-1.24	.001
Countdown × Predictable × PSWQ × MASQ	-0.00	0.00	-0.00, 0.00	660	-1.83	.003
Countdown × Unpredictable × PSWQ × MASQ	0.00	0.00	0.00, 0.01	660	2.52	.005*
**Random Components**						

σ^2	10.93
τ_{00}	32.38
ICC	.75
Marginal R^2	.092
Conditional R^2	.771
$N_{participants}$	136
Observations	816

Note. For contrast coded conditions, neutral is the reference category, for contrast coded cues, interstimulus interval is the reference category. PSWQ = Penn-State Worry Questionnaire (grand-mean centered); MASQ = Mood and Anxiety Questionnaire (Anxious Arousal subscale, grand-mean centered); σ^2 = residual variance; τ_{00} = random intercept; ICC = Intra class correlation;
*** $p < .001$; ** $p < .01$; * $p < .05$

Supplement D: Full multilevel model for P3

Table S4

Multilevel model predicting startle probe locked P3 in µV to the NPU-Threat test.

**Fixed Effects**	***b***	***SE***	**95% *CI***	***df***	***t***	R^2
Intercept	4.75	0.33	4.10, 5.39	132	14.36	***
Countdown	0.29	0.12	0.06, 0.52	660	2.48	.006*
Predictable	-0.98	0.17	-1.31, -0.66	660	-5.91	.030***
Unpredictable	0.07	0.17	-0.26, 0.39	660	0.41	.000
PSWQ	0.02	0.03	-0.03, 0.07	132	0.76	.004
MASQ	-0.06	0.05	-0.15, 0.03	132	-1.40	.014
Countdown × Predictable	0.38	0.17	0.05, 0.70	660	2.28	.005*
Countdown × Unpredictable	-0.31	0.17	-0.64, 0.02	660	-1.87	.003
Countdown × PSWQ	0.01	0.01	-0.01, 0.03	660	1.12	.001
Countdown × MASQ	-0.02	0.02	-0.06, 0.01	660	-1.48	.002
Predictable × PSWQ	0.03	0.01	0.00, 0.05	660	2.03	.004*
Predictable × MASQ	-0.01	0.02	-0.06, 0.03	660	-0.54	.000
Unpredictable × PSWQ	-0.01	0.01	-0.03, 0.02	660	-0.66	.000
Unpredictable × MASQ	-0.02	0.02	-0.06, 0.03	660	-0.82	.001
PSWQ × MASQ	-0.00	0.00	-0.01, 0.00	132	-0.98	.007
Predictable × PSWQ × MASQ	0.00	0.00	-0.00, 0.00	660	1.12	.001
Unpredictable × PSWQ × MASQ	0.00	0.00	-0.00, 0.00	660	0.22	.000
Countdown × Predictable × PSWQ	-0.00	0.01	-0.03, 0.02	660	-0.35	.000
Countdown × Predictable × MASQ	-0.03	0.02	-0.07, 0.01	660	-1.31	.002
Countdown × PSWQ × MASQ	0.00	0.00	-0.00, 0.00	660	0.61	.000
Countdown × Unpredictable × PSWQ	-0.02	0.01	-0.04, 0.01	660	-1.32	.002
Countdown × Unpredictable × MASQ	0.06	0.02	0.02, 0.11	660	2.81	.007*
Countdown × Predictable × PSWQ × MASQ	0.00	0.00	-0.00, 0.00	660	0.54	.000
Countdown × Unpredictable × PSWQ × MASQ	-0.00	0.00	-0.00, 0.00	660	-0.51	.000

**Random Components**	
σ^2	10.02
τ_{00}	11.57
ICC	.54
Marginal R^2	.061
Conditional R^2	.564
$N_{participants}$	136
Observations	816

Note. For contrast coded conditions, neutral is the reference category, for contrast coded cues, interstimulus interval is the reference category. PSWQ = Penn-State Worry Questionnaire (grand-mean centered); MASQ = Mood and Anxiety Questionnaire (Anxious Arousal subscale, grand-mean centered); σ^2 = residual variance; τ_{00} = random intercept; ICC = Intra class correlation;
*** $p < .001$; ** $p < .01$; * $p < .05$

To test the specificity of this effect to PSWQ we exploratorily included BDI-II into the model, which did not change the pattern of results. Specifically, the interaction (Predictable × PSWQ) remained significant (b = -0.03, SE = 0.01, $t(789)$ = 2.03, p = .042).

Supplement E: *t*-transformed startle analyses

Of note, the random-intercept model failed to converge with *t*-transformed startle amplitudes. The data were *t*-transformed within each individual, so the mean value for each participant was 50 (*SD* = 10). Hence, the *t*-transformation decreased the random variance in the data and thus a generalized linear model was estimated.

Table S5

Generalized linear regression models predicting t-transformed startle responses to the NPU-Threat test.

**Fixed Effects**	***b***	***SE***	**95% *CI***	***t***	***p***
Intercept	49.99	0.15	49.79, 50.28	339.02	<.001
Countdown	-0.15	0.15	-0.43, 0.14	-0.99	.323
Predictable	-2.21	0.21	-2.62, -1.80	-10.58	<.001
Unpredictable	3.95	0.21	3.54, 4.36	18.97	<.001
PSWQ	0.00	0.01	-0.02, 0.02	0.06	.955
MASQ	-0.00	0.02	-0.04, 0.04	-0.03	.974
Countdown × Predictable	-0.84	0.21	-1.25, -0.43	-4.00	.001
Countdown × Unpredictable	0.01	0.21	-0.40, 0.42	0.04	.970
Countdown × PSWQ	0.01	0.01	-0.01, 0.04	1.07	.283
Countdown × MASQ	-0.00	0.02	-0.04, 0.04	-0.17	.866
Predictable × PSWQ	-0.00	0.02	-0.04, 0.03	-0.21	.833
Predictable × MASQ	-0.05	0.03	-0.10, 0.01	-1.62	.106
Unpredictable × PSWQ	0.06	0.02	0.03, 0.09	3.62	.003
Unpredictable × MASQ	0.06	0.03	-0.00, 0.11	1.93	.053
PSWQ × MASQ	0.00	0.00	-0.00, 0.00	0.02	.981
Predictable × PSWQ × MASQ	-0.00	0.00	-0.00, 0.00	-0.03	.978
Unpredictable × PSWQ × MASQ	-0.00	0.00	-0.00, 0.00	-0.94	.348
Countdown × Predictable × PSWQ	-0.00	0.02	-0.04, 0.03	-0.24	.813
Countdown × Predictable × MASQ	-0.01	0.03	-0.07, 0.04	-0.45	.656
Countdown × PSWQ × MASQ	-0.00	0.00	-0.00, 0.00	-0.77	.440
Countdown × Unpredictable × PSWQ	0.04	0.02	0.01, 0.07	2.43	.015

Countdown × Unpredictable × MASQ	-0.01	0.03	-0.06, 0.05	-0.28	.781
Countdown × Predictable × PSWQ × MASQ	0.00	0.00	-0.00, 0.00	0.16	.872
Countdown × Unpredictable × PSWQ × MASQ	-0.00	0.00	-0.00, 0.00	-0.56	.574

**Model fit**	$\boldsymbol{R^2}$	***F***	***df***	***p***
	.091	19.9	23, 4577	<.001

Note. For contrast coded conditions, neutral is the reference category, for contrast coded cues, interstimulus interval is the reference category. PSWQ = Penn-State Worry Questionnaire (grand-mean centered); MASQ = Mood and Anxiety Questionnaire (Anxious Arousal subscale, grand-mean centered);

Supplement F: Range-corrected startle analyses

Table S6

Multilevel model predicting range-corrected startle responses to the NPU-Threat test. To calculate the range-corrected amplitudes, each raw amplitude of a participant was divided by that participant's maximal amplitude and multiplied by 100 so that all amplitudes lie between 0 and 100.

Fixed Effects	*b*	*SE*	95% *CI*	*df*	*t*	R^2
Intercept	21.95	1.14	19.72, 24.17	132	19.32	***
Countdown	-0.41	0.38	-1.14, 0.33	4424	-1.09	.000
Predictable	-5.51	0.53	-6.56, -4.47	4424	-10.37	.025***
Unpredictable	9.87	0.53	8.83, 10.91	4424	18.62	.076***
PSWQ	0.09	0.09	-0.09, 0.26	132	0.95	.002
MASQ	-0.18	0.16	-0.49, 0.12	132	-1.18	.003
Countdown × Predictable	-2.19	0.53	-3.23, -1.15	4424	-4.11	.004***
Countdown × Unpredictable	0.09	0.53	-0.95, 1.13	4424	0.17	.000
Countdown × PSWQ	0.03	0.03	-0.03, 0.09	4424	0.95	.000
Countdown × MASQ	-0.01	0.05	-0.11, 0.09	4424	-0.18	.000
Predictable × PSWQ	-0.02	0.04	-0.10, 0.06	4424	-0.50	.000
Predictable × MASQ	-0.11	0.07	-0.25, 0.03	4424	-1.48	.001
Unpredictable × PSWQ	0.16	0.04	0.07, 0.24	4424	3.74	.003***
Unpredictable × MASQ	0.14	0.07	0.00, 0.29	4424	1.99	.001*
PSWQ × MASQ	0.01	0.01	-0.00, 0.03	132	1.62	.006
Predictable × PSWQ × MASQ	0.00	0.00	-0.01, 0.01	4424	0.09	.000
Unpredictable × PSWQ × MASQ	-0.01	0.01	-0.02, 0.01	4424	-1.08	.000
Countdown × Predictable × PSWQ	-0.01	0.04	-0.10, 0.07	4424	-0.34	.000
Countdown × Predictable × MASQ	-0.03	0.07	-0.17, 0.12	4424	-0.36	.000
Countdown × PSWQ × MASQ	-0.00	0.00	-0.01, 0.00	4424	-0.62	.000
Countdown × Unpredictable × PSWQ	0.10	0.04	0.02, 0.18	4424	2.37	.001*
Countdown × Unpredictable × MASQ	-0.02	0.07	-0.16, 0.12	4424	-0.26	.000
Countdown × Predictable × PSWQ × MASQ	0.00	0.00	-0.01, 0.01	4424	0.38	.000
Countdown × Unpredictable × PSWQ × MASQ	-0.00	0.00	-0.01, 0.00	4424	-0.85	.000
**Random Components**						

σ^2	572.69
τ_{00}	139.29
ICC	.20
Marginal R^2	.078
Conditional R^2	.258
$N_{participants}$	136
Observations	4578

Note. For contrast coded conditions, neutral is the reference category, for contrast coded cues, interstimulus interval is the reference category. PSWQ = Penn-State Worry Questionnaire (grand-mean centered); MASQ = Mood and Anxiety Questionnaire (Anxious Arousal subscale, grand-mean centered); σ^2 = residual variance; τ_{00} = random intercept; ICC = Intra class correlation;
*** $p < .001$; ** $p < .01$; * $p < .05$;

Supplement G: Power Simulations

The power analysis was computed using simr version 1.0.5 (Green & Macleod, 2016). Each analysis is based on 1,000 Monte Carlo simulations (Arend & Schäfer, 2019). For each outcome (i.e., startle, N1, P3), we simulated the power of the consistent highest order effect along the sample size. The resulting power estimates and power curves (Figure S1) indicate the likelihood of finding comparable true effects in the data given the sample size of n = 101.

In the startle data, the consistent effect across analytical approaches was the cross-level interaction PSWQ × condition. According to the result of the power simulation, we could have detected comparable effects in the data with a likelihood of 90.10 % (95 % CI [88.08, 91.88]).

For N100, we simulated the level 1 effect of condition, as this was the only emergent effect in the data. The simulation result suggests that the power for finding comparable level 1 effects in the N100 data was 100 % (95 % CI [99.63, 100.0]).

For P300, we simulated the cross-level interaction of PSWQ × condition. The result indicates that comparable interaction effects in the P300 data could have been found with a probability of 70.10 % (95 % CI [67.16, 72.92]).

Figure S1 Power curves using 1,000 Monte Carlo simulations for the relevant effects of each outcome. Error bars depict the 95 % CI.

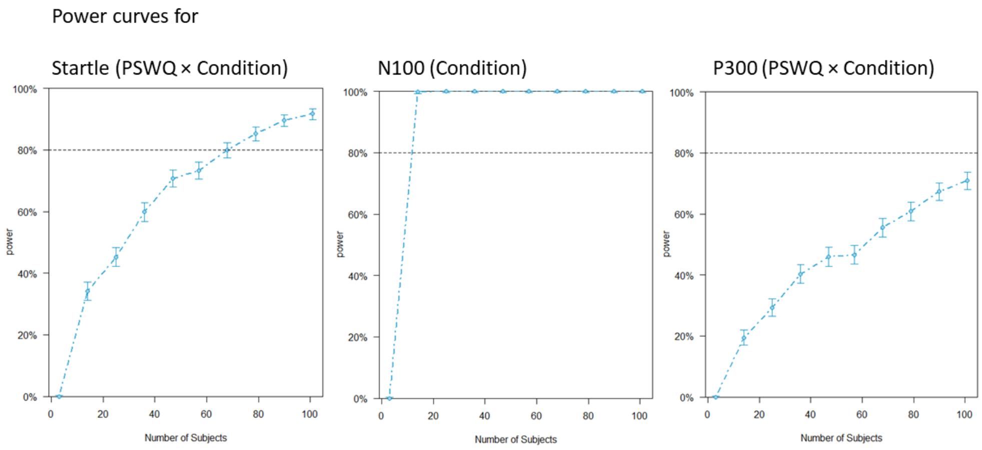

Supplement H: Grand averages

Figure S2 Grand averages and topographical depictions of the event-related potentials N1 and P3 collapsed across experimental conditions

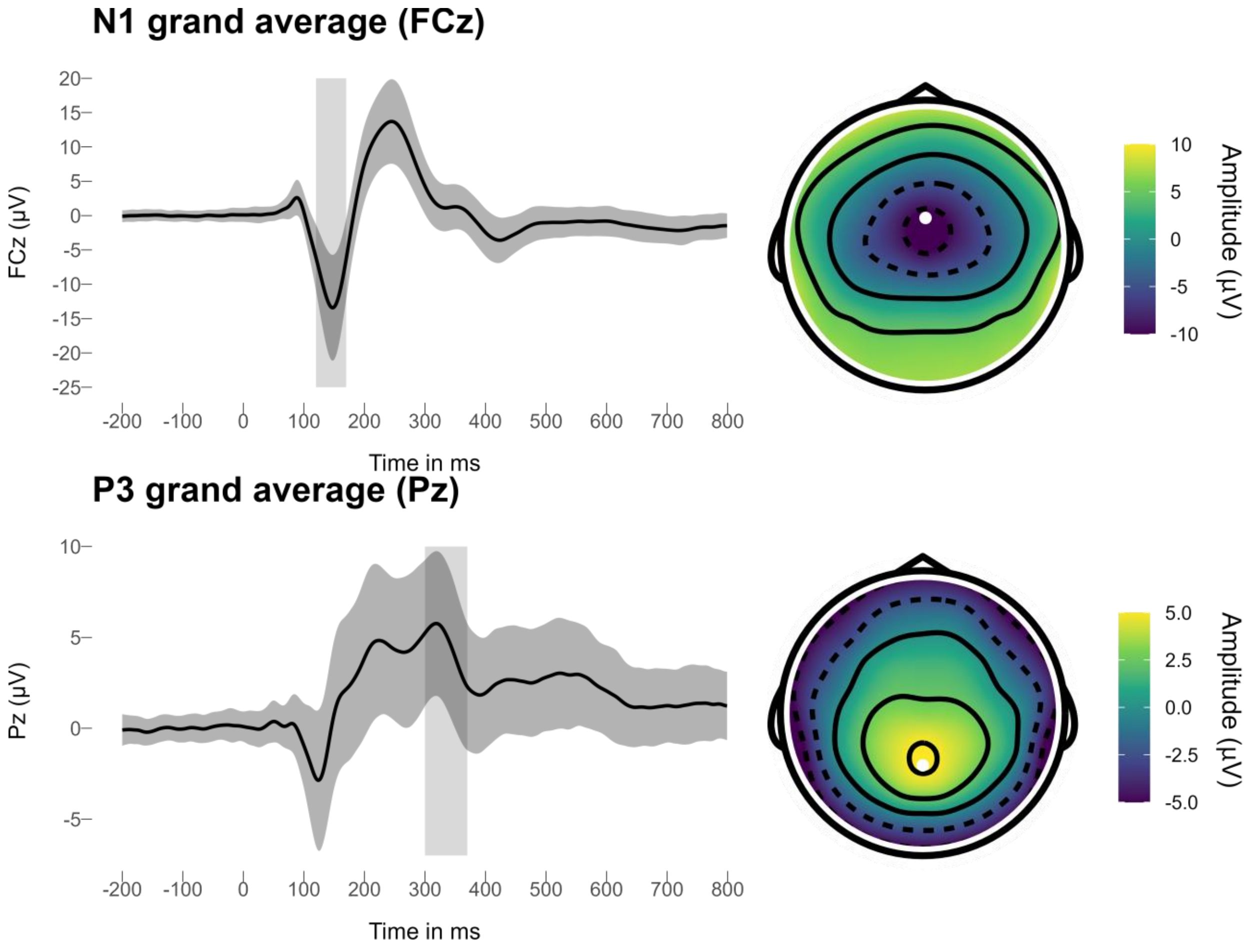

Note. The signal is locked to startle probes and is collapsed across conditions and cues of the NPU-Threat test. The time windows for quantification of N1 (± 25 ms area around the individual peak at FCz; white dot; ca. 120-170 ms) and P3 (mean activity 300 – 370 ms at Pz; white dot) are indicated by shaded grey bars. The shaded area around the mean signal depicts ± 1 standard deviation of the distribution at each ms.
